# Intimate Partner Violence Perpetration Among Sexual Minority People and Associated Factors: A Systematic Review of Quantitative Studies

**DOI:** 10.1007/s13178-022-00761-4

**Published:** 2022-09-08

**Authors:** Tommaso Trombetta, Luca Rollè

**Affiliations:** Department of Psychology, University of Turin, Via Verdi 10, 10124 Torino, TO Italy

**Keywords:** Intimate partner violence, IPV, IPV perpetration, Sexual minority people, Systematic review, Quantitative studies

## Abstract

**Introduction:**

Intimate partner violence (IPV) among sexual minority people has been underestimated since few decades ago despite its spreading. The current systematic review aims to review and systematize studies on factors associated with IPV perpetration within this population.

**Methods:**

Data search was conducted on EBSCO and PubMed considering articles published until July 2022, and 78 papers were included.

**Results:**

Although methodological limitations can affect the results found, the data demonstrated an association between IPV perpetration and psychological, relational, family of origin-related and sexual minority-specific factors, substance use, and sexual behaviors.

**Conclusion:**

The findings emerged highlight the importance of a multidimensional approach to tackle IPV perpetration among sexual minority people and limit relapses, while increasing individual and relational wellbeing.

**Policy Implications:**

The empirical evidence emerged can contribute to the development of policies and services tailored for sexual minority people victims of IPV, to date still scarce and often ineffective.

## Introduction

Couple violence suffered and perpetrated by sexual minority people^1^ was largely understudied until a few decades ago (Kimmes et al., 2019). In contrast, research and public opinion focused primarily on violence within heterosexual couples, influencing and being influenced by a mainstream heteronormative discourse on couple violence mainly focused on violent men who abuse their female partner because of a patriarchal and sexist culture that justify these behaviors as expression of masculinity (Rollè et al., 2020, 2021).

Nevertheless, many studies demonstrated rates of IPV among sexual minority people that are comparable, if not higher, than those identified among heterosexual couples (e.g., Walters et al., 2013; West, 2012). Establishing firm conclusions regarding the prevalence of IPV among same-sex couples seems particularly complex because of methodological limitations (e.g., lack of generalizable data, differences in the operationalization of IPV) and differences between research (Rollè et al., 2018, 2019). In addition, studies with large or representative samples have been limited and mainly conducted in US states, while data from European countries are still lacking and other research in this direction are needed. However, a representative study by Walters et al. (2013) showed alarming results: nearly one-third of sexual minority men and one-half of sexual minority women in the USA reported having suffered psychological or physical IPV in their lifetime. In addition, no significant differences emerged in the prevalence of IPV between lesbian and heterosexual women, and gay and heterosexual men (Walters et al., 2013). A meta-analysis by Badenes-Ribera et al. (2015) confirmed these results among lesbian women, finding a mean lifetime prevalence of IPV victimization of 48%.

Despite the widespread prevalence of this phenomenon, few research has been conducted on IPV among sexual minority people. A study by Edwards et al. (2015) found that only 400 (approximately 3%) out of the 14,200 studies published between 1999 and 2013 that addressed couple violence examined participants with a non-heterosexual orientation.

Although attention on couple violence among sexual minority people has increased in the last decades, the data available are still scarce, and influenced by methodological limitations. For example, most researches have used convenience samples and are cross-sectional in nature. Differences in the operative definitions of violence and sexual orientation emerged as well and make it difficult to compare results and draw firm conclusions about characteristics, antecedents, and consequences of couple violence in sexual minority people (Mason et al., 2014; Murray & Mobley, 2009).

Many similarities have been found between IPV in sexual minorities and heterosexual people such as the cycle of violence (Messinger, 2011; Walker, 1979; Whitton et al., 2019), the forms of suffered abuse (i.e., physical, psychological, sexual, and controlling violence, and unwanted pursuit), and some of the associated factors — for example, relationship satisfaction (Balsam & Szymanski, 2005), mental health (Sharma et al., 2021), personality (Landolt & Dutton, 1997), adult attachment (Bartholomew et al., 2008a, b; Gabbay & Lafontaine, 2017b), family-of-origin violence (Fortunata & Kohn, 2003), and substance abuse (Wei et al., 2020a, b).

However, peculiarities of IPV among sexual minority people emerged as well. Specifically, as highlighted in the minority stress model proposed by Meyer (1995, 2003), sexual minority people suffer particular adverse conditions (i.e., experiences of discrimination, perceived stigma, internalized homonegativity, and sexual identity concealment) that affect their individual and relational wellbeing (e.g., Hughes et al., 2022; Pachankis et al., 2021; Pachankis et al., 2018), and which seem to increase the risk to suffer or perpetrate IPV (Edwards et al., 2015; Rollè et al., 2018).

In addition, sexual minority people are affected by some specific forms of abuse: threats of outing to significant others and homonegative attitudes expressed toward the partner emerged as specific abusive tactics acted out by sexual minority persons (Badenes-Ribera et al., 2016).

Furthermore, the help-seeking process within this population is influenced by unique complexities. According to several authors (Calton et al., 2016; Cannon & Buttell, 2015; Chong et al., 2013; Ollen et al., 2017; Rollè et al., 2021), the heteronormative and homonegative climate that still permeates our societies limits the opportunity of understanding, recognizing, and managing this phenomenon. The lack of services tailored to this population and the ineffectiveness of formal sources of support have been extensively documented (Freeland et al., 2018; Lorenzetti et al., 2017; Rollè et al., 2021; Santoniccolo et al., 2021). This negatively influences the possibilities of sexual minority people who are victims or perpetrators of IPV to find help and recover from this experience.

Given similarities and differences between IPV in sexual minorities and heterosexual people, and the negative consequences this phenomenon has on victims’ physical (e.g., injuries, risk of suicidality) and psychological (e.g., symptoms of depression, anxiety, and stress) wellbeing (Bartholomew et al., 2008a, b; Robinson, 2002; Strickler & Drew, 2015), understanding what variables are associated with the perpetration of IPV among sexual minority people can provide important information for clinical purposes.

Accordingly, the current paper aims to review and systematize the scientific literature focused on the exploration of factors associated to the perpetration of IPV among sexual minority people. Many studies have highlighted the lack of interventions tailored to sexual minority people who experience IPV as well as the ineffectiveness of mainstream formal sources of support, partly due by the lack of knowledge about LGBT+-related themes and specificities of IPV among sexual minorities people (see Santoniccolo et al., 2021 for a review on this topic). The implementation of policies and services capable of addressing the complexities and specificities experienced by sexual minority people involved in couple violence is still needed (Subirana-Malaret et al., 2019). Data obtained in the current review can provide empirical evidence in this direction, providing an exhaustive summary of the current knowledge on the phenomenon, which can guide the development of future prevention and intervention programs addressed to sexual minority people who perpetrate couple violence. Furthermore, the current paper aims to highlight limitations and gaps of the current literature and provide insights for future research.

## Materials and Methods

### Data Source and Search Strategy

The current systematic review followed the Preferred Reporting Items for Systematic Review and Meta-Analyses (PRISMA) statement (Moher et al., 2009; Page et al., 2021). Two independent reviewers (TT and LR) conducted a systematic search through EBSCO (Databases: APA Psycinfo; CINAHL Complete; Family Studies Abstracts; Gender Studies Database; Race Relations Abstracts; Social Sciences Abstracts [H.W. Wilson]; Sociology Source Ultimate; Violence & Abuse Abstracts) and PubMed. No temporal limits were imposed on the search. All the articles published from the beginning of the databases to July 2022 were screened.

The following keywords were applied: violence or abuse or aggression or batter* AND partner or couple* or domestic or intimate or dating AND “same-sex” or “same-gender” or gay or lesbian* or bisex* or lgb* or homosexual* or “m*n who ha* sex with m*n” or msm or “wom*n who ha* sex with wom*n” or wsw or “m*n who ha* sex with m*n and wom*n” or msmw or “wom*n who ha* sex with wom*n and m*n” or wswm or “sexual minorit*” or “m*n who love m*n” or “wom*n who love wom*n”.

### Inclusion and Exclusion Criteria

The following inclusion criteria were applied to select the studies: (a) original research papers, (b) published in peer-review journals, (c) in the English language, (d) focused on the assessment of factors associated with the perpetration of IPV among sexual minority people (i.e., self-identified LGB+ people, people sexually or romantically attracted to people of the same-sex, people involved in same-sex relationship or people that reported non-heterosexual sexual behaviors); (e) only quantitative studies were eligible for the inclusion.

All the studies that did not match the inclusion criteria reported above were excluded. In addition, the following exclusion criteria were applied: (a) studies pertaining to IPV whose methods or results did not clearly differentiate between IPV among sexual minority people and heterosexual people; (b) validation studies, meta-analyses and literature reviews; (c) qualitative studies; (d) papers focused only on factors associated to IPV victimization among sexual minority people; (e) papers that assessed factors associated with any form of IPV (regardless of victim or perpetrator status) among sexual minority people which, however, did not differentiate between variables related to perpetration and those related to victimization. These studies were excluded because they do not provide clear information on factors associated with the perpetration of IPV, and thus do not provide data which can guide the development of interventions targeted to perpetrators. Finally, (f) articles mainly focused on transpeople or self-identified heterosexual people perpetrators of IPV were excluded. However, some of the studies included in the current systematic review involved small percentages of gender minorities or self-identified heterosexual people that based on their sexual behaviors or romantic attraction were classified as sexual minority people. These studies were retained because, from our perspective, they still provide data that can inform on factors related to IPV perpetration among cisgender sexual minority people, which was the population of our interest.

### Study Selection and Data Extraction

The search through EBSCO returned 5956 articles, and 4028 papers were left after duplicates removal. Of these, 414 papers were selected for full-text review after the screening of title and abstract, and 73 papers were included. PubMed provided 2174 articles in total. After the screening of title and abstract, 216 papers were selected for full-text review. The removal of duplicates between databases left 143 articles and five were included. In total, 78 articles were included in the current systematic review after full-text reading and the application of inclusion and exclusion criteria.

**Fig. 1:**
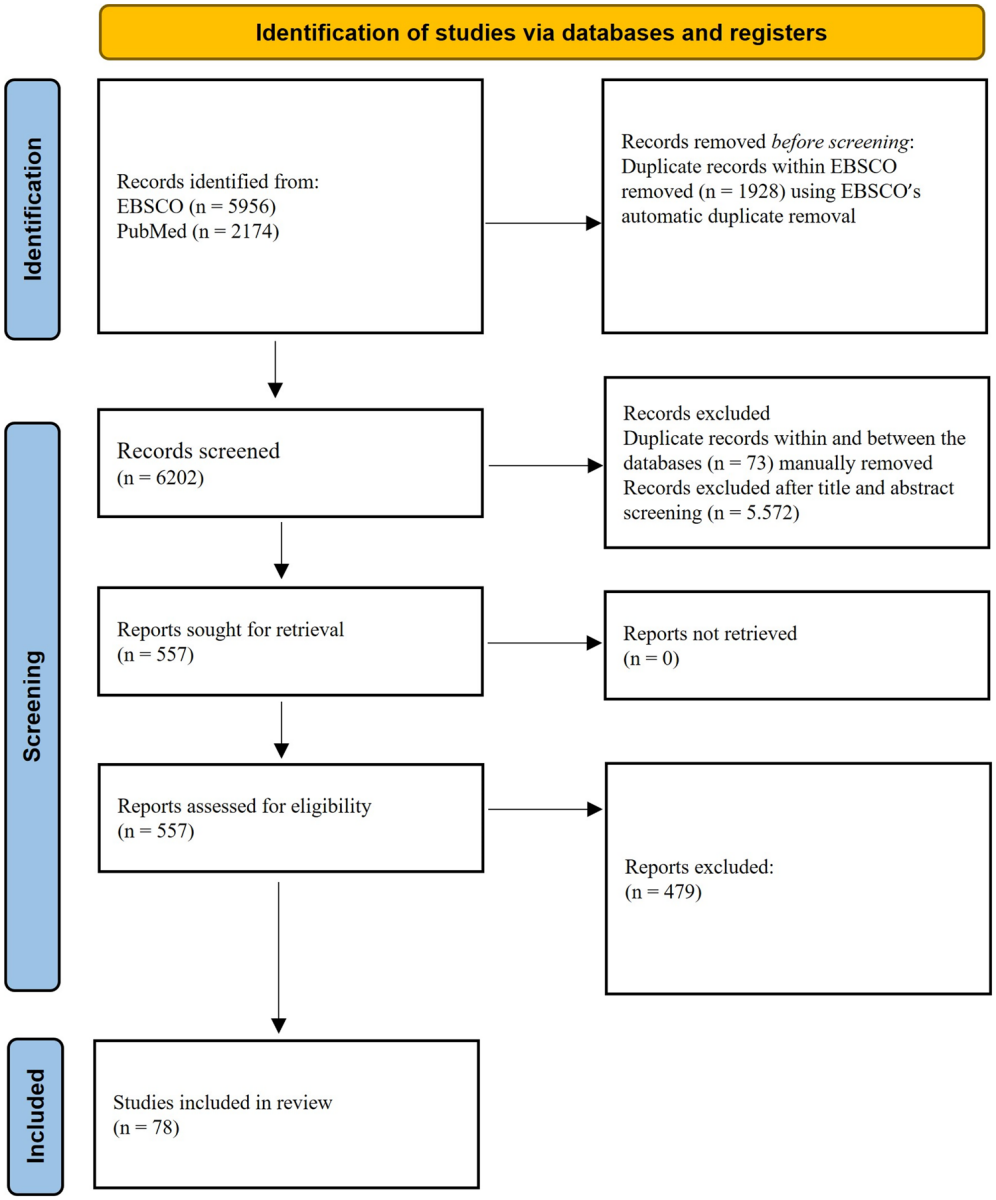
Flowchart of the selection procedure

Two independent reviewers analyzed the full-text and proceeded with the data extraction. Any disagreement was discussed between the reviewers in order to obtain a unanimous consensus. See Fig. 1 for a summary of the study selection procedure.

## Results

Seventy-eight studies were included in the current systematic review and most of them (54 studies) were conducted in the USA. Two studies were conducted in Canada, and two in the USA and Canada. Nine studies were conducted in Europe: three in Italy, two in England, one in Germany, one in Turkey and one in Spain; one study was in Turkey and Denmark. Five studies were conducted in China, one in Spanish-speaking countries (Spain, Mexico, Chile, and Venezuela), one in Latin American countries (mainly in Mexico), one in Puerto Rico, one in South Africa, and one in Hong Kong. Finally, one study assessed factors associated with IPV perpetration in the USA, Canada, Australia, UK, South Africa, and Brazil (see Table 2).

The last two decades have seen an increase in the number of articles published on the topic of our interest. Specifically, while three studies were published between 1988 and 1999, and 15 between 2000 and 2010, most were published between 2011 and 2021 (60 studies; see Table 2).

### Methodological Issues

Several methodological differences between the included studies emerged, which must be accounted for when considering the obtained results.

First, differences in the operationalization of violence and in the used instruments were found. Thirty-nine studies assessed IPV perpetration with some version of the conflict tactics scale (CTS; Ayhan Balik & Bilgin, 2021; Bartholomew et al., 2008a, b; Causby et al., 1995; Balsam & Szymanski, 2005; Chong et al., 2013; Craft & Serovich, 2005; Craft et al., 2008; Edwards & Sylaska, 2013; Edwards et al., 2021; Leone et al., 2022; Lewis et al., 2017; Li et al., 2019, 2022; Li & Zheng, 2021; Gabbay & Lafontaine, 2017a; Gabbay & Lafontaine, 2017b; Jones & Raghavan, 2012; Kelley et al., 2014; Landolt & Dutton, 1997; Lewis et al., 2018; Mason et al., 2016; McKenry et al., 2006; Milletich et al., 2014; Oringher & Samuelson, 2011; Pepper & Sand, 2015; Pistella et al., 2022; Stephenson et al., 2011b; Stults et al., 2021b; Swan et al., 2021; Swann et al., 2021; Taylor & Neppl, 2020; Tognasso et al., 2022; Whitton et al., 2021; Wu et al., 2015; Stults et al., 2015b; Stults et al., 2016; Waterman et al., 1989; Whitton et al., 2019; Zavala, 2017). Other used assessment tools were the Intimate Partner Violence Among Gay and Bisexual Men (IPV-GBM) Scale (10 studies; Davis et al., 2016; Jones & Raghavan, 2012; Sharma et al., 2021; Stephenson & Finneran, 2016; Stephenson & Finneran, 2017; Suarez et al., 2018; Wei et al., 2020a, b, 2021; Zhu et al., 2021); the Psychological Maltreatment of Women Inventory (three studies; Bartholomew et al., 2008a, b; Leone et al., 2022; Lewis et al., 2018) or its short form (one study; Lewis et al., 2017); the 30-item Abusive Behavior Inventory (ABI; two studies; Telesco, 2003; Turell et al., 2018); the Multidimensional Measure of Emotional Abuse (two studies; Fontanesi et al., 2020; Ummak et al., 2021); the Cold Violence Scale (one study; Li & Zheng, 2021); the SGM-Specific IPV Tactics Scale (one study; Swann et al., 2021); the Conflict in Adolescent Dating and Relationships Inventory (one study; Reuter et al., 2015); the Relational Pursuit-Pursuer Short Form (one study; Derlega et al., 2011); the Sexual Coercion in Intimate Relationships Scale (one study; Fontanesi et al., 2020); the Psychological Maltreatment Inventory (one study; Landolt & Dutton, 1997); the Sexual Experiences Survey (one study; Krahé et al., 2000); the Perpetration in Dating Relationships (PDR; one study; Jacobson et al., 2015); The Safe Dates—Psychological Abuse Perpetration scale (one study; Jacobson et al., 2015); the 19 items of the Psychological Abuse in Intimate Partner Violence Scale adapted to be used with same-sex couples (EAPA-P; Longares et al., 2018a; one study; Longares et al., 2018a); the Fundamental Interpersonal Relations Orientation-Behavior (FIRO-B; one study; Poorman & Seelau, 2001); The Coercive Control Scale (one study; Whitton et al., 2019); SGM-Specific IPV Tactics Scale (one study; Whitton et al., 2019); the Cyber Abuse Scale (one study; Whitton et al., 2019); an adaptation of the scale developed by Smith et al., (1995; one study; Wong et al., 2010). In contrast, 19 studies (Bacchus et al., 2017; Bartholomew et al., 2008a, b; Bogart et al., 2005; Carvalho et al., 2011; Finneran & Stephenson, 2014; Finneran et al., 2012; Kelly et al., 2011; Li & Zheng, 2021; Longares et al., 2018b; Messinger et al., 2021; Miltz et al., 2019; Reuter et al., 2015; Schilit et al., 1991; Stephenson et al., 2011a, 2013; Stults et al., 2015a, 2021a; Toro-Alfonso & Rodríguez-Madera, 2004; Waterman et al., 1989) used items developed by authors to assess at least some forms of IPV perpetration (see Table 2 for more details).

Second, only 15 studies (Balsam & Szymanski, 2005; Davis et al., 2016; Edwards et al., 2021; Fortunata & Kohn, 2003; Jones & Raghavan, 2012; Sharma et al., 2021; Stephenson & Finneran, 2016; Stephenson & Finneran, 2017; Suarez et al., 2018; Swann et al., 2021; Wei et al., 2020a, b; Whitton et al., 2019; Whitton et al., 2021; Zhu et al., 2021) included instruments or items that addressed abusive tactics specific to sexual minority people (e.g., threats of outing, homonegativity, and negative HIV-related attitudes). Furthermore, only slightly more than half of the studies were focused on the variables associated to the violence perpetrated within a same-sex relationship (40 studies; Ayhan Balik & Bilgin, 2021; Balsam & Szymanski, 2005; Bartholomew et al., 2008a, b; Bartholomew et al., 2008a, b; Carvalho et al., 2011; Chong et al., 2013; Craft & Serovich, 2005; Davis et al., 2016; Edwards & Sylaska, 2013; Fontanesi et al., 2020; Gabbay & Lafontaine, 2017a; Gabbay & Lafontaine, b; Jacobson et al., 2015; Jones & Raghavan, 2012; Kahle et al., 2020; Kelley et al., 2014; Landolt & Dutton, 1997; Leone et al., 2022; Lewis et al., 2017; Lewis et al., 2018; Li et al., 2019, 2022; Li & Zheng, 2021; Longares et al., 2018b; Mason et al., 2016; Milletich et al., 2014; Pepper & Sand, 2015; Pistella et al., 2022; Poorman & Seelau, 2001; Schilit et al., 1990; Sharma et al., 2021; Stephenson & Finneran, 2016; Stephenson et al., 2011a; Stephenson et al., 2011a, b; Stephenson et al., 2013; Suarez et al., 2018; Telesco, 2003; Tognasso et al., 2022; Wu et al., 2015), while the remaining articles did not specify in what kind of relationship the violence occurred (i.e., if within a relationship with a same-sex or opposite-sex partner).

Third, differences in the characteristics of the involved population emerged. Only one study used probabilistic methods of sampling (Bogart et al., 2005). Most (39 studies) included self-identified LGB+ people (Ayhan Balik & Bilgin, 2021; Bacchus et al., 2017; Bartholomew et al., 2008a, b; Bartholomew et al., 2008a, b; Bogart et al., 2005; Chong et al., 2013; Derlega et al., 2011; Edwards et al., 2021; Finneran & Stephenson, 2014; Finneran et al., 2012; Fontanesi et al., 2020; Fortunata & Kohn, 2003; Gabbay & Lafontaine, 2017a; Gabbay & Lafontaine, b; Jacobson et al., 2015; Kelly et al., 2011; Lewis et al., 2017; Lewis et al., 2018; Longares et al., 2018a; Longares et al., b; Mason et al., 2016; Oringher & Samuelson, 2011; Pistella et al., 2022; Poorman & Seelau, 2001; Reuter et al., 2015; Schilit et al., 1990; Sharma et al., 2021; Stephenson & Finneran, 2016; Stephenson & Finneran, 2017; Stephenson et al., 2011a; Stephenson et al., 2013; Swan et al., 2021; Taylor & Neppl, 2020; Telesco, 2003; Tognasso et al., 2022; Toro-Alfonso & Rodríguez-Madera, 2004; Turell et al., 2018; Ummak et al., 2021; Zavala, 2017). Regardless of their self-identified sexual orientation, 17 studies recruited participants on the basis of their involvement in a same-sex relationship (Balsam & Szymanski, 2005; Craft & Serovich, 2005; Craft et al., 2008; Edwards & Sylaska, 2013; Jones & Raghavan, 2012; Kahle et al., 2020; Kelley et al., 2014; Leone et al., 2022; Li et al., 2019, 2022; Li & Zheng, 2021; Milletich et al., 2014; Pepper & Sand, 2015; Stephenson et al., 2011a, b; Suarez et al., 2018; Waterman et al., 1989; Wu et al., 2015), while twelve articles were focused on people who reported non-heterosexual sexual behaviors (Davis et al., 2016; Krahé et al., 2000; Miltz et al., 2019; Stults et al., 2015a, b, 2016, 2021a, b; Wei et al., 2020a, b, 2021; Zhu et al., 2021). In five studies, participants were recruited based on one of the criteria reported above (i.e., sexual orientation, same-sex relationship, sexual behaviors; Messinger et al., 2021; Swann et al., 2021; Whitton et al., 2019, 2021; Wong et al., 2010). Five articles did not specify based on what criteria they defined their participants as sexual minority people (Carvalho et al., 2011; Causby et al., 1995; McKenry et al., 2006; Landolt & Dutton, 1997; Schilit et al., 1991). In terms of age, education and ethnicity of the enrolled participants, most of the studies (54 studies) included in the current systematic review were focused on adult participants, mainly White and well-educated (Ayhan Balik & Bilgin, 2021; Bacchus et al., 2017; Balsam & Szymanski, 2005; Bartholomew et al., 2008a, b; Bartholomew et al., 2008a, b; Bogart et al., 2005; Carvalho et al., 2011; Chong et al., 2013; Derlega et al., 2011; Edwards & Sylaska, 2013; Edwards et al., 2021; Finneran et al., 2012; Fontanesi et al., 2020; Fortunata & Kohn, 2003; Gabbay & Lafontaine, 2017a; Gabbay & Lafontaine, b; Jacobson et al., 2015; Kahle et al., 2020; Kelley et al., 2014; Kelly et al., 2011; Krahé et al., 2000; Landolt & Dutton, 1997; Leone et al., 2022; Lewis et al., 2017; Lewis et al., 2018; Li & Zheng, 2021; Li et al., 2022; Longares et al., 2018a; Longares et al., b; Mason et al., 2016; McKenry et al., 2006; Milletich et al., 2014; Miltz et al., 2019; Oringher & Samuelson, 2011; Pepper & Sand, 2015; Pistella et al., 2022; Schilit et al., 1990, 1991; Sharma et al., 2021; Stephenson & Finneran, 2017; Stephenson et al., 2011a; Stephenson et al., 2011a, b; Suarez et al., 2018; Swan et al., 2021; Taylor & Neppl, 2020; Tognasso et al., 2022; Toro-Alfonso & Rodríguez-Madera, 2004; Turell et al., 2018; Ummak et al., 2021; Waterman et al., 1989; Wei et al, 2020a, b, 2021; Zhu et al., 2021). Only five studies involved adolescents (Poorman & Seelau, 2001; Reuter et al., 2015; Stults et al., 2015b, 2016; Whitton et al., 2019), three were mainly focused on HIV-positive participants (Bogart et al., 2005; Craft & Serovich, 2005; Wu et al., 2015), and in 20 articles, ethnic minorities or ethnically diverse people were the population of interest (Craft & Serovich, 2005; Craft et al., 2008; Davis et al., 2016; Finneran & Stephenson, 2014; Jones & Raghavan, 2012; Li et al., 2019, Messinger et al., 2021; Reuter et al., 2015; Stephenson & Finneran, 2016; Stephenson et al., 2013; Stults et al., 2015a, b, 2016, 2021a, b; Swann et al., 2021; Whitton et al., 2021; Wong et al., 2010; Wu et al., 2015; Zavala, 2017). The study by Causby et al. (1995) did not specify the characteristics of the included participants.

Finally, while the characteristic of both partners can influence and being influenced by IPV perpetration, only eleven studies used dyadic techniques of data analysis (Landolt & Dutton, 1997; Leone et al., 2022; Lewis et al., 2018; Li et al., 2019, 2022; Schilit et al., 1990; Sharma et al., 2021; Stephenson et al., 2011a, b; Stephenson et al., 2013; Suarez et al., 2018; Turell et al., 2018), and only two adopted a longitudinal design (Lewis et al., 2018; Stults et al., 2021a), while the remaining 76 articles were cross-sectional in nature.

### Main Findings

The following classes of variables (see Table 1 for a definition of each class of variables) were identified as factors associated to the perpetration of IPV among sexual minority people: sociodemographic factors; psychological factors; relational factors; social and community-level factors; feminine and masculine gender expression; intimate partner violence; family of origin-related factors; substance use; medical conditions; sexual behaviors; sexual minorities-specific factors (see Table 2 for a summary of the results found within the studies included and their methodological characteristics).

**Table 1:** Definition of the IPV-associated factors analyzed in the results section

Factor	Definition
Socio-demographic factors	Social and cultural factors that characterize a specific person/population
Psychological factors	Personality traits, mental representations and functioning, and mental health symptoms
Relational factors	Characteristics of the relationship (i.e., duration, monogamy, cohabitation), dyadic satisfaction or adjustment and conflict resolution skills, communication and power dynamics
Social- and community-level factors	Characteristics of the social network and perceived social support
Feminine and masculine gender expression, and sexism	Behavioral or personal characteristics consistent with masculine or feminine gender stereotypes, and negative attitudes based on sex
Intimate partner violence	Forms of violence experienced and episodes of IPV victimization
Family of origin-related factors	Previous experiences of violence (witnessed or suffered) or harsh parenting within the family of origin
Substance use	Alcohol and drug use and abuse
Medical conditions	Sexually transmitted infections and PrEP use
Sexual behaviors	Years at anal sexual debut, number of partners, characteristics of sexual intercourses, and unprotected sex
Sexual minority–specific factors	Minority stressors (i.e., experiences of discriminations, perceived stigma, internalized homonegativity, and outness) and gay identity development

**Table 2:** Characteristics of the included studies

Author(s)	Title	Country	Participants	Form(s) of IPV assessed	Assessment tool(s)	Conclusions
Ayhan Balik and Bilgin (2021)	Experiences of minority stress and intimate partner violence among homosexual women in Turkey	Turkey	*N* = 149 (F)	Psychological, physical, sexual IPV, and injury	CTS2	Outness was positively associated to physical, psychological, and sexual IPV perpetration (not to injury). Discrimination was not associated to IPV perpetration. Internalized homonegativity was positively associated only to sexual IPV perpetration
Bacchus et al. (2017)	Occurrence and impact of domestic violence and abuse in gay and bisexual men: a cross sectional survey	England	*N* = 532(M)	Negative behaviors towards the partner, defined as frightening him/her; demanding that the partner asks for permission to work, go shopping, visit relatives or visit friends; physical violence; sexual coercion	Survey developed by the authors based on Comparing Heterosexual and Same Sex Abuse in Relationships (COHSAR) survey	Depression was not associated with IPV perpetration. There was a marginal association between symptoms of mild anxiety disorder and any negative behaviors in the past 12 months, while symptoms of mild anxiety disorder were not associated with physical abuse, frightening, forcing sex, or controlling behaviors. Alcohol use was not associated with IPV perpetration. Alcohol dependence or abuse was not associated with IPV perpetration. Participants who reported frightening and physically hurting their partner were at increased risk of cannabis use compared to those who did not; there were no differences in cannabis use between those who perpetrate forcing sex or any abusive behaviors in the past 12 months, or those whose partner need to ask permission to doing activities, and those who did not; physically hurting a partner, but no other forms of abuse, was related with class A drugs use. Perpetrators of any abusive behaviors in the past 12 months were at lower risk of having a diagnosis of sexually transmitted infections (STI) than non-perpetrators; perpetrators of physical abuse, frightening, forcing sex, or controlling behaviors did not differ from those who did not perpetrate these forms of violence in the risk of having an STI diagnosis
Balsam and Szymanski (2005)	Relationship quality and domestic violence in women's same-sex relationship: the role of minority stress	USA	*N* = 272 (F)	Any IPV (physical and sexual); verbal IPV; LGB-specific tactics of psychological aggression	Conflict Tactics Scale, Revised Edition (CTS2) to assess physical/sexual and verbal IPV; 5 items developed by authors concerning LGB-specific tactics of psychological aggression	Age was not associated with IPV perpetration. Education was negatively associated with lifetime physical and sexual IPV, but not with recent IPV. Income was not associated with IPV perpetration. Dyadic adjustment was negatively associated with IPV perpetration. Gender expression was not associated with IPV perpetration. Lifetime discrimination was associated with psychological and physical/sexual (not LGB-specific abuse) IPV perpetration, while past-year discrimination was not. Experiences of discrimination were not associated with IPV perpetration. Outness was not related to IPV perpetration. Internalized homonegativity was positively associated with physical/sexual IPV, and this relation was fully mediated by dyadic adjustment; internalized homonegativity was not related to psychological IPV and LGB-specific abuse
Bartholomew et al. (2008)	Correlates of partner abuse in male same-sex relationships	Canada	*N* = 186 (M)	Psychological and physical abuse	A modified version by Bartholomew et al., (2008a, b) of the Conflict Tactics Scales (CTS)	Age was not associated with IPV perpetration. Less educated people were at increased risk of IPV perpetration, however, when controlling for IPV victimization this association was no longer significant. Income was negatively associated with physical, but not emotional, IPV perpetration; however, this relation was no longer significant when controlling for IPV victimization. Attachment anxiety was associated with IPV perpetration; however, only attachment anxiety assessed through interview, and not self-reported anxious attachment, was still associated with physical and psychological IPV perpetration when controlling for IPV victimization; attachment avoidance was associated with IPV perpetration, however, only attachment avoidance assessed through interview was associated with physical and psychological IPV perpetration, while self-reported attachment avoidance was not. IPV victimization was associated with IPV perpetration. Witnessing violence in the family of origin was not associated with IPV perpetration. Childhood maltreatment was positively associated with IPV perpetration. Alcohol and drug use were both positively associated with IPV perpetration; however, these relations were no longer significant when controlling for IPV victimization. HIV status was not related to IPV perpetration. Outness was positively related to IPV perpetration when controlling for internalized homonegativity, though this relation became non-significant when controlling for both internalized homonegativity and violence receipt. Internalized homonegativity was positively associated with IPV perpetration
Bartholomew et al., (2008a, b)	Patterns of abuse in male same-sex relationships	Canada	*N* = 284 (M)	Physical abuse; psychological abuse; sexual abuse; physical injury	CTS to assess physically abusive acts; 13 items derived from the latest version of the CTS and the Psychological Maltreatment of Women Inventory both used to assess psychological abuse; 7 items developed by the authors, of which 2 used to assess sexual abuse and 5 used to assess physical injury	IPV victimization was associated with IPV perpetration. Psychological, sexual, and psychological IPV, and physical injury were associated with each other, however, the association between physical IPV and physical injury was no longer significant when controlling for IPV victimization
Bogart et al. (2005)	The association of partner abuse with risky sexual behaviors among women and men with HIV/AIDS	USA	*N* = 726, 286 (F), 440 (M)	Any IPV (threats to hurt the partner, physical violence, and sexual coercion)	8 items developed by the authors	Unprotected sex was associated with IPV perpetration
Carvalho et al. (2011)	Internalized sexual minority stressors and same-sex intimate partner violence	USA	*N* = 567, 262 (F), 305 (M)	Any IPV	1 item developed by the authors	Perceived stigma was positively associated with IPV perpetration. Neither outness nor internalized homonegativity were related to IPV perpetration
Causby et al. (1995)	Fusion and conflict resolution in lesbian relationships	USA	*N* = 275 (F)	Verbal aggression, physical aggression, physical violence	CTS	Self-esteem was negatively associated with IPV perpetration. Share fusion was associated with physical aggression, physical/more severe violence, and psychological violence, while time fusion was only associated with physical aggression and psychological violence
Chong et al. (2013)	Risk and protective factors of same-sex intimate partner violence in Hong Kong	Hong Kong	*N* = 306, 192 (F), 114 (M)	Psychological aggression and physical assault	CTS2	No gender differences in IPV perpetration were found; Age was not associated with IPV perpetration. Sexual orientation was not associated with IPV perpetration. Less educated people were at increased risk of IPV perpetration. Income was negatively associated with physical, but not sexual, IPV perpetration. Anger management was negatively associated with physical and psychological IPV perpetration, and the relation between anger management and physical IPV perpetration was fully mediated by psychological IPV. Self-efficacy was not associated with IPV perpetration. Cohabitation with a same-sex partner was not associated with physical or psychological IPV perpetration. Length of relationship was not associated with IPV perpetration. Dominance was positively associated with IPV perpetration; however, this relation was no longer significant when controlling for demographic variables. Relationship conflict was positively associated with physical and psychological IPV. Physical and psychological aggressions were positively correlated. Substance use (i.e., both alcohol and other drugs use) was positively associated with physical, but not psychological, IPV perpetration. Internalized homonegativity was not associated with IPV perpetration
Craft and Serovich (2005)	Family-of-origin factors and partner violence in the intimate relationships of gay men who are HIV positive	USA	*N* = 51 (M)	Physical assault, psychological aggression, sexual coercion, physical injury	CTS2	Witnessing violence from mother-to-father was positively associated with sexual coercion, while witnessing violence from father-to-mother was not. Witnessing violence (both from mother-to-father and from father-to-mother) was not associated with psychological IPV, physical assault or physical injury perpetration
Craft et al. (2008)	Stress, attachment style, and partner violence among same-sex couples	USA	*N* = 87, 46 (M), 41 (F)	Psychological aggression; physical aggression; sexual coercion	CTS2	No gender differences in IPV perpetration were found. Perceived stress was positively associated with IPV perpetration, and this relation was fully mediated by insecure attachment
Davis et al. (2016)	Associations between alcohol use and intimate partner violence among men who have sex with men	USA	*N* = 189 (M)	Physical and sexual, monitoring, controlling, HIV related-IPV, and emotional violence + a total score (any IPV)	IPV-GBM Scale	Alcohol use was associated with physical/sexual and emotional IPV toward both regular and casual partner, and with controlling and HIV-related IPV perpetration toward regular, but not casual, partner; monitoring IPV perpetration was not associated with alcohol use
Derlega et al. (2011)	Unwanted pursuit in same-sex relationships: effects of attachment styles, investment model variables, and sexual minority stressors	USA	*N* = 153, 84 (F), 66 (M), 3 (unidentified)	UPB perpetration; aggressive behaviors	The 28-item Relational Pursuit–Pursuer Short Form Questionnaire	Men engaged in more pursuit behaviors than women did. No gender differences were found regarding aggressive behaviors. Attachment anxiety was associated with perpetration of pursuit behaviors, but not with aggressive behaviors; attachment avoidance was not associated with pursuit or aggressive behaviors. Relationship satisfaction was not associated with perpetration of pursuit or aggressive behaviors. Higher scores in investment size, not in poor quality of alternatives or commitment in relationships, were related to perpetration of unwanted pursuit behaviors (and not with aggressive behaviors). Frequency of minority stressors experienced was not associated with perpetration of pursuit behaviors or negative behaviors
Edwards et al. (2021)	Minority stress and sexual partner violence victimization and perpetration among LGBQ + college students: the moderating roles of hazardous drinking and social support	USA	*N* = 1221, 885 (F), 175 (M), 119 (gender queer, gender nonconforming, or nonbinary), 32 (transgender), 4 (other), 6 (not disclosed)	Sexual IPV	SGM-CTS2	IPV perpetration was positively related to IPV victimization. IPV perpetration was unrelated to problem drinking, minority stress, or social support. Minority stress (identity concealment; internalized homonegativity; stigma consciousness) was not related to perpetrating IPV. Problem drinking moderated the relation between minority stress and IPV perpetration: among those with higher levels of problem drinking, minority stress was associated with a higher likelihood of perpetration, while this relation was not significant at low levels of problem drinking. Social support did not moderate this relation
Edwards and Sylaska (2013)	The Perpetration of Intimate Partner Violence among LGBTQ College Youth: The Role of Minority Stress	USA	*N* = 391, 191 (M), 178 (F), 18 (genderqueer), 4 (other)	Physical, sexual and psychological abuse	CTS2	Experiences of discrimination were not associated with IPV perpetration. Internalized homonegativity was associated with physical and sexual IPV perpetration, but not with psychological IPV
Finneran and Stephenson (2014)	Intimate partner violence, minority stress, and sexual risk-taking among U.S. men who have sex with men	USA	*N* = 1575(M)	Physical and sexual IPV	2 items developed by the authors	Age was not associated with IPV perpetration. Sexual orientation was not associated with IPV perpetration. Ethnicity was not associated with IPV perpetration. Education was negatively associated with physical, but not sexual, IPV perpetration. Employment was not associated with IPV perpetration. Sexual IPV perpetration was associated with psychological, but not physical, IPV perpetration; psychological IPV perpetration was associated with physical IPV perpetration. HIV status was not related to IPV perpetration. Perpetrators of physical IPV were more likely to have had unprotected anal intercourse than non-perpetrators of physical IPV; no differences in unprotected anal intercourse emerged between perpetrators of sexual IPV and non-perpetrators of sexual IPV; physical IPV, but not sexual IPV, was higher among participants who have had unprotected anal intercourses (UAI) compared with those who have not had. Homophobic discrimination was positively associated with IPV perpetration in the ANOVA test, while this relation was not significant in the logistic model. Internalized homonegativity was associated with sexual IPV, but not with physical IPV, perpetration
Finneran et al. (2012)	Intimate partner violence and social pressure among gay men in six countries	USA, Canada, Australia, the UK, South Africa, and Brazil	*N* = 2368 (M)	Physical and sexual violence	2 items developed by the authors	Age was associated with sexual IPV perpetration only in USA (participants aged between 25 and 34 were at increased risk of IPV) and Australia (participants older than 34 were at increased risk of IPV), and not in Canada, Brazil, South Africa, UK. Education was associated with IPV perpetration only in Canada: those who had more than 12 years of education were less likely to perpetrate IPV. Internalized homonegativity was positively associated with IPV only in United Kingdom. Ethnicity, HIV status, drug use, behavioral bisexuality, homophobic discrimination, and heteronormativity were not associated with IPV
Fontanesi et al. (2020)	The role of attachment style in predicting emotional abuse and sexual coercion in gay and lesbian people: an explorative study	Italy	*N* = 182, 106 (F), 76 (M)	Emotional abuse (restrictive engulfment, denigration, hostile withdrawal, and dominance/intimidation); sexual coercion	The Multidimensional Measure of Emotional Abuse to assess emotional abuse; The Sexual Coercion in Intimate Relationship Scale to assess sexual coercion	No gender differences in IPV perpetration were found. Confidence was negatively associated with commitment defection and manipulation, but not with coercion of resources and violence; a positive association was found between confidence and acted emotional abuse; discomfort with closeness was negatively associated with coercion of resources and violence, but was not related to commitment defection, manipulation, and acted emotional abuse; need for approval was negatively related to coercion of resources and violence, and manipulation, but not with commitment defection or acted emotional abuse; preoccupation with relationship was positively related to commitment defection, and negatively associated to coercion of resources and violence, manipulation, and acted emotional abuse; relationship being secondary was not associated with sexual coercion or acted emotional abuse
Fortunata and Kohn (2003)	Demographic, psychosocial, and personality characteristics of lesbian batterers	USA	*N* = 100 (F); perpetrators = 38, non-perpetrators = 62	Physical violence	The CTS-L (Lesbian): Conflict Tactics Scale (CTS; Straus, 1979) as modified by Coleman (1991) for lesbian couples	Ethnicity was not associated with IPV perpetration. Batterers’ partners had lower income than non-batterers' partners. Employment was not associated with IPV perpetration. Batterers had higher scores on the aggressive (sadistic), antisocial, avoidant, passive-aggressive, self-defeating, borderline, paranoid, and schizotypal personality scale scores and higher alcohol-dependent, drug-dependent, bipolar (manic syndrome), and delusional clinical syndrome scale scores; however, no significant differences between batterers and non-batterers emerged in the scores on compulsive, dependent, depressive, histrionic, narcissistic, schizoid, anxiety, dysthymia, PTSD, somatoform, major depression, and thought disorders scales; when controlling for desirability and debasement, group differences for the avoidant, bipolar (manic syndrome), dependent, passive-aggressive, schizoid, schizotypal, and self-defeating personality were no longer significant. Having a child was not associated with IPV perpetration. Batterers and non-batterers did not differ regarding monogamous relationships. Childhood maltreatment was positively associated with IPV perpetration. There were no differences between abusers and non-abusers in having a family member during childhood who abused substances. Alcohol use, alcohol dependence or abuse, and drug use were positively associated with IPV perpetration
Gabbay and Lafontaine (2017a)	Do trust and sexual intimacy mediate attachment’s pathway toward sexual violence occurring in same sex romantic relationships?	Canada and USA	*N* = 310, 107 (M), 203 (F)	Sexual violence	CTS2	Attachment anxiety was associated with sexual IPV perpetration; this association was fully mediated by dyadic trust and sexual intimacy in a serial mediation model; attachment avoidance was associated with sexual IPV perpetration, and this relation was partially mediated by dyadic trust and sexual intimacy in a serial mediation model
Gabbay and Lafontaine (2017b)	Understanding the relationship between attachment, caregiving, and same sex intimate partner violence	Canada and USA	*N* = 310, 107 (M), 203 (F)	Psychological and physical violence	CTS2	No gender differences in IPV perpetration were found. Attachment anxiety was not associated with IPV perpetration; attachment avoidance was associated with physical, but not psychological, IPV perpetration, and this relation was no longer significant when controlling for receipt of violence; the proximity dimension of caregiving (and not sensitivity, compulsive caregiving and controlling caregiving) was negatively associated with physical and psychological IPV perpetration, although this relation was not significant when controlling for receipt of violence; a significant association was found between psychological IPV perpetration and both hyperactivation of the attachment and caregiving systems and deactivation of the attachment and caregiving systems, even in the presence of each other. Regarding physical IPV perpetration, only hyperactivation was still associated with physical couple violence when controlling for the effect of deactivation strategies. None of these findings were significant when receipt of violence was controlled for. IPV victimization was associated with IPV perpetration
Jacobson et al. (2015)	Gender expression differences in same-sex intimate partner violence victimization, perpetration, and attitudes among LGBTQ college students	USA	*N* = 278, 115 (M), 163 (F)	Any IPV (physical and sexual violence); psychological abuse	The Perpetration in Dating Relationships (PDR) to assess physical and sexual violence; The Safe Dates—Psychological Abuse Perpetration scale (SD-PAP) to assess psychological abuse	Masculinity was positively associated with IPV perpetration
Jones and Raghavan (2012)	Sexual orientation, social support networks, and dating violence in an ethnically diverse group of college students	USA	*N* = 114, 60 (M), 54 (F)	Physical dating violence; sexual dating violence	CTS2	Being involved in a male network composed by perpetrators of violence was positively associated with dating or sexual violence only among lesbian women and not among gay men
Kahle et al. (2020)	The influence of relationship dynamics and sexual agreements on perceived partner support and benefit of PrEP use among same-sex male couples in the U.S	USA	*N* = 659 (M)	Any IPV (physical, sexual, monitoring, controlling and emotional)	IPV-GBM Scale	IPV perpetrators did not differ with non-perpetrators in thinking their partner would not support their PrEP use or in not knowing if their partner would support their PrEP use, or in their perception of benefits provided by PrEP use
Kelley et al. (2014)	Predictors of perpetration of men’s same-sex partner violence	USA	*N* = 107 (M)	Physical violence	CTS2	Alcohol use was associated with IPV perpetration, and this relation was moderated by outness: only at high levels of outness, this association was significant. IPV perpetrators have lower levels of outness compared with non-perpetrators. Internalized homonegativity was positively associated with IPV perpetration
Kelly et a;. (2011)	The intersection of mutual partner violence and substance use among urban gays, lesbians, and bisexuals	USA	*N* = 2200, 1782 (M), 418 (F)	Physical and non-physical (verbal threats, property destruction) violence	1 measure developed by the authors	Alcohol and drug use were not associated with IPV perpetration
Krahé et al. (2000)	Ambiguous communication of sexual intentions as a risk marker of sexual aggression	Germany	study 1: *N* = 526, 283 (F), 243 (M); study 2: *N* = 454, 173 (F), 281 (M)	Sexual aggression	The Sexual Experiences Survey (SES)	Token resistance was positively associated with sexual violence, while the association between sexual violence and compliance was not significant
Landolt and Dutton (1997)	Power and personality: an analysis of gay male intimate abuse	USA	*N* = 52 same-sex couples	Physical abuse; emotional abuse	CTS to assess physical abuse; Psychological Maltreatment Inventory (PMI), used to assess emotional abuse	Both actor’s and partner’s abusive personality was associated with physical and psychological IPV perpetration; more specifically, each constituent of the abusive personality of both the actor and the partner were associated with psychological IPV perpetration; for physical IPV perpetration both actor and partner effects were significant for BPO, fearful, and preoccupied attachment, while neither actor nor partner effects were significant for anger and paternal rejection, and only actor effects were significant for maternal rejection. Both actor’s and partner’s abusive personality was associated with physical and psychological IPV perpetration; more specifically, each constituent of the abusive personality of both the actor and the partner were associated with psychological IPV perpetration; for physical IPV perpetration both actor and partner effects were significant for BPO, fearful, and preoccupied attachment, while neither actor nor partner effects were significant for anger and paternal rejection, and only actor effects were significant for maternal rejection., perpetration of psychological IPV by abusers was higher when victims perceive to be in a divided-power couple compared when victims perceived to be in an egalitarian couple. No other differences regarding psychological IPV perpetration emerged when comparing victims’ perception of being in a divided-power, egalitarian or self-dominant couple; couples that disagree in their perception of relationship power dynamics (i.e., non-congruent couples) did not differ from congruent couple in their levels of IPV perpetration
Leone et al. (2022)	A dyadic examination of alcohol use and intimate partner aggression among women in same-sex relationships	USA	*N* = 163 couples (F)	Physical and psychological IPV	CTS2, PMWI	Participants’ drink per week (DPW) consumed were positively related to one's own physical, but not psychological, IPV perpetration. Partner's DPW was associated with participants’ psychological, but not physical, IPV perpetration. Participants’ hazardous alcohol use was associated with both physical and psychological IPV perpetration, while partner's hazardous drink use was associated only to psychological IPV perpetration
Lewis et al. (2017)	Empirical investigation of a model of sexual minority specific and general risk factors for intimate partner violence among lesbian women	USA	*N* = 1048 (F)	Psychological aggression (dominance-isolation (e.g., jealousy, treating as an inferior, and isolation from resources) and emotional-verbal violence (e.g., name calling, screaming, and swearing); physical violence	The 28-item short form of the Psychological Maltreatment of Women Inventory (PMWI) to assess psychological aggression; the 12 physical assault items from the CTS2 to assess physical violence	While physical violence perpetration and victimization were each other associated, only the association between psychological IPV perpetration and psychological IPV victimization was significant, but the opposite directional path was not. A complex relation between discrimination, internalized homonegativity, perpetrator trait anger, perpetrator’s and partner’s alcohol problems, perpetrator’s relationship dissatisfaction, and psychological and physical violence
Lewis et al. (2018)	Discrepant drinking and partner violence perpetration over time in lesbians’ relationships	USA	*N* = 1052(F)	Physical assault; psychological maltreatment	12 items from the physical assault subscale and six items from the Injury subscale of the CTS2 for physical aggression; the short form of the PMWI for psychological aggression	Physical aggression was associated with discrepant drinking between partners at a later time point, while discrepant drinking was not related to subsequent physical aggression; discrepant drinking was associated with subsequent psychological aggression and vice versa
Li et al. (2019)	Internalized homophobia and relationship quality among same-sex couples: the mediating role of intimate partner violence	USA	*N* = 144 same-sex couples	Physical IPV; psychological IPV	The Conflict Tactics Scale-Couple Form Revised (CTS-CF-R)	Relationship satisfaction was negatively associated with psychological, but not physical, IPV perpetration. Participants’ and partner’s internalized homonegativity were associated with psychological, but not physical, IPV
Li et al. (2022)	Sexual minority stressors and intimate partner violence among same-sex couples: commitment as a resource	USA	*N* = 144 couples, 109 (F), 35 (M)	Physical and psychological IPV	The Conflict Tactics Scale-Couple Form Revised (CTS-CF- R; Straus et al., 1996)	Internalized homophobia and discrimination were positively associated to IPV perpetration, while commitment in the relationship was negatively associated to IPV perpetration. Commitment moderated the association between internalized homonegativity and partner's (not participants’) psychological (not physical) IPV perpetration: when commitment was high, this association was no longer significant. No other moderating effects of commitment on the association between internalized homonegativity and participants’ or partner’s IPV perpetration were found. Own’s commitment (not partner's commitment) moderated the association between own’s discrimination (not partner's discrimination), and participants’ and partner’s psychological IPV perpetration prevalence and frequency: at high levels of commitment these relations were no longer significant. No other moderating effects of own’s or partner’s commitment in the relation between own’s or partner’s discrimination and participants’ or partner’s IPV perpetration prevalence or frequency were found. Individuals’ (not partner's) internalized homophobia was positively related to a higher frequency (not prevalence) of individuals’ own and the partner’ psychological (not physical) IPV perpetration through lower levels individuals own’ commitment (not through partner's commitment). Individuals’ discrimination (not partner’s discrimination) was negatively related to frequency (not prevalence) of individual's own and the partner’s psychological (not physical) IPV perpetration through higher levels of partner’s commitment (not individual's own commitment). Individuals’ (not partner’s) internalized homophobia was positively related to individual’s own and the partner’s physical (not psychological) IPV perpetration through lower levels of individual’s own commitment. Individuals’ (not partner’s) discrimination was negatively related to lower likelihood of individuals’ own and the spouses’ physical (not psychological) IPV perpetration through higher levels of partner’s (not individual's own) commitment. No other mediating effects of commitment on IPV perpetration were detected
Li & Zheng (2021)	Intimate partner violence and controlling behavior among male same-sex relationships in China: relationship with ambivalent sexism	China	*N* = 272 (M)	Psy, phys, sex, inj; cold violence (economic and personal control, emotional and sexual negligence); dominance, emotional control, financial control, intimidation, social/isolation, and threats	CTS2S; Cold Violence Scale; 34-item scale designed by the researchers	Number of sexual partner was positively related only to perpetration of emotional negligence, emotional control, and threats, but not to psychological, physical, sexual IPV, and injury, economic and personal control, dominance, financial control, intimidation, social/isolation. Both benevolent and hostile sexism toward women was positively associated to Cold Violence perpetration, but not to IPV or controlling behaviors perpetration. Hostile attitudes toward men were positively related only to controlling behaviors perpetration, while Hostile sexism toward men was not associated to IPV perpetration, cold violence, or controlling behaviors
Longares et al. (2018a)	Insecure attachment and perpetration of psychological abuse in same-sex couples: a relationship moderated by outness	Spanish-speaking people who were mostly residents in Spain (44.26%), Mexico (20%), Chile (8.5%), and Venezuela (8.5%)	*N* = 305, 157 (M), 148 (F)	Psychological abuse	Adaptation of the 19 items on the Psychological Abuse in Intimate Partner Violence Scale (EAPA-P)	Insecure adult attachment was associated with psychological IPV; outness moderated this relation: at low levels of overall outness, the relationship between insecure attachment and psychological IPV was not significant; similarly, at low and high levels of outness to religion this association was not significant; outness to the family did not moderate the association between insecure attachment and psychological IPV. Overall outness, and not outness to religion and outness to the family, was positively related to psychological IPV perpetration
Longares et al. (2018b)	Psychological abuse in Spanish same-sex couples: prevalence and relationship between victims and perpetrators	Spain	*N* = 107, 54 (M), 53 (F)	Psychological abuse	Items developed by authors	No gender differences in IPV perpetration were found. IPV victimization was associated with IPV perpetration
Mason et al. (2016)	Minority stress and intimate partner violence perpetration among lesbians: negative affect, hazardous drinking, and intrusiveness as mediators	USA	*N* = 342 (F)	Physical IPV	CTS2	A complex relation between general life stress, distal and proximal minority stressors, negative affect, hazardous alcohol use, intrusiveness, and physical IPV perpetration was detected
McKenry et al. (2006)	Perpetration of gay and lesbian partner violence: a disempowerment perspective	USA	*N* = 77, 40 (M), 37 (F)	Physical violence	CTS2	Non-perpetrating females reported higher psychological adjustment compared with non-perpetrating males, and male and female perpetrators. IPV perpetrators experienced more family stress than non-perpetrators. Self-esteem was negatively associated with IPV perpetration. Perpetrators have less secure attachment style than non-perpetrators. Relationship satisfaction was not associated with IPV perpetration. Dependence was not related to IPV perpetration. Perceived power differentials were not associated with IPV perpetration. Masculinity was positively associated with IPV perpetration. Witnessing violence in the family of origin was not associated with IPV perpetration. Perpetrators of IPV grew up in lower SES families than non-perpetrators. Alcohol use was positively associated with IPV perpetration. Internalized homonegativity was positively associated with IPV perpetration
Messinger et al. (2021)	Sexual and gender minority intimate partner violence and childhood violence exposure	USA	*N* = 457 (FAB SGM)	Physical and psychological violence	Two items developed by the authors	Older and Black and Latin participants were more likely to perpetrate IPV than younger and White participants. Parental verbal and physical IPV were positively associated to psychological e physical IPV. Childhood sexual abuse was related only to physical IPV perpetration. To witness violence between siblings was positively associated only to psychological IPV perpetration; to witness parental violence to both physical and psychological IPV. Gender of perpetrator of violence in the family of origin was not associated to IPV perpetration
Milletich et al. (2014)	Predictors of women’s same-sex partner violence perpetration	USA	*N* = 209 (F)	Physical violence	CTS2	Less educated people were at increased risk of IPV perpetration. Fusion was positively associated with IPV perpetration. Dominance/accommodation was not directly associated with IPV perpetration; however, an indirect relation mediated by fusion was found between these variables. Witnessing violence in the family of origin was not associated with IPV perpetration. Internalized homonegativity was not directly related to IPV perpetration, while there was a positive indirect association between these variables that was mediated by fusion
Miltz et al. (2019)	Intimate partner violence, depression, and sexual behavior among gay, bisexual and other men who have sex with men in the PROUD trial	England	*N* = 436 (M)	Any IPV (psychological, physical and sexual)	10 items developed by the authors	Sexual orientation was not associated with IPV perpetration. Ethnicity was not associated with IPV perpetration. Less educated people were at increased risk of IPV perpetration. Employment was not associated with IPV perpetration. Depression was positively associated with IPV perpetration. Drug use during sex was associated with IPV perpetration. Years at anal sexual debut and number of sexual partners were not associated with IPV. perpetration. Having group sex was associated with perpetration of lifetime, but not past year, IPV. Unprotected sex was not associated with IPV perpetration. Outness was not related to IPV perpetration. Internalized homonegativity was positively associated with IPV perpetration
Oringher and Samuelson (2011)	Intimate partner violence and the role of masculinity in male same-sex relationships	USA	*N* = 117 (M)	Physical assault, sexual coercion and injury	CTS2	A positive association was found between physical IPV victimization and physical IPV perpetration, and between sexual IPV victimization and sexual IPV perpetration; sexual and physical IPV perpetration were positively associated. Several dimensions of masculinity were associated with physical IPV perpetration: suppression of vulnerability and aggressiveness were both positively related to physical IPV perpetration, while avoidance of dependency on other was negatively related to physical IPV perpetration; the association between self-destructive achievement and dominance, and physical IPV perpetration was not significant; no dimensions of masculinity were associated with sexual IPV perpetration
Pepper and Sand (2015)	Internalized homophobia and intimate partner violence in young adult women’s same-sex relationships	USA	*N* = 40 (F)	Physical aggression, psychological assault, sexual coercion, injury	CTS2	Psychological maladjustment was positively associated with psychological, but not physical or sexual, IPV perpetration. Hostility was positively associated with IPV perpetration. Emotional instability was positively related to physical and psychological IPV perpetration, but not with sexual IPV. Negative worldview was associated with psychological, but not physical or sexual, IPV perpetration. Emotional unresponsiveness was not associated with IPV perpetration. Negative self-esteem and self-adequacy were not associated with IPV perpetration. Dependence was not related to IPV perpetration. Physical IPV victimization was associated with physical IPV perpetration; psychological IPV victimization was associated with psychological IPV perpetration; sexual IPV victimization was not associated with sexual IPV perpetration. Sexual coercion perpetration was associated only with the religious attitudes toward Lesbianism dimension of the Lesbian Internalized Homonegativity Scale (LIHS), while it was not related to any other dimension of the LIHS. Internalized homonegativity was not related to physical and emotional IPV perpetration
Pistella et al. (2022)	Psychosocial impact of Covid-19 pandemic and same-sex couples conflict: the mediating effect of internalized sexual stigma	Italy	*N* = 232, 131 (F), 101 (M)	Any IPV	CTS2S	Couple conflict and IPV victimization were positively related to IPV perpetration; sexual satisfaction was negatively related to IPV perpetration. Psychosocial impact of COVID-19, age, internalized sexual stigma, relationship duration, religiosity, and involvement in LGB associations were not related to IPV perpetration
Poorman and Seelau (2001)	Lesbians who abuse their partners: using the FIRO-B to assess interpersonal characteristics	USA	*N* = 15 (F)	Psychological abuse	The Fundamental Interpersonal Relations Orientation-Behavior (FIRO-B)	Perpetrators had lower expressed and wanted inclusion, and expressed and wanted affection compared with non-perpetrators; However, expressed and wanted control did not differ between perpetrators and non-perpetrators, and there were no differences between the groups in the differences between expressed and wanted inclusion, expressed and wanted affection or expressed and wanted control
Reuter et al. (2015)	An exploratory study of teen dating violence in sexual minority youth	USA	*N* = 782, 444 (M), 338 (F)	Physical, psychological, sexual, and relational violence	The Conflict in Adolescent Dating and Relationship Inventory (CADRI)	No gender differences in IPV perpetration were found. Sexual orientation was associated with severe, and not any, TDV perpetration. Hostility was positively associated with IPV perpetration. Social support was not related to IPV perpetration. Witnessing violence in the family of origin was not associated with IPV perpetration. Alcohol use was not associated with IPV perpetration
Schilit et al. (1990)	Substance use as a correlate of violence in intimate lesbian relationships	USA	*N* = 107 (F)	Any IPV (sexual, physical and emotional)	A questionnaire developed by the authors	Participants’ alcohol use, but not partner's alcohol use, was positively associated with IPV perpetration. Participants’ and partner's drug use were not associated with IPV perpetration
Schilit et al. (1991)	Intergenerational transmission of violence in lesbian relationships	USA	*N* = 104 (F)	Sexual, verbal-emotional and physical abuse	A 70-items questionnaire developed by the authors	Witnessing intimate partner violence in the family of origin was positively associated with IPV perpetration. Childhood maltreatment was positively associated with IPV perpetration
Sharma et al. (2021)	Sexual agreements and intimate partner violence among male couples in the U.S.: an analysis of dyadic data	USA	*N* = 386 same-sex couples	Physical, sexual, monitoring, controlling and emotional IPV	IPV-GBM Scale	Depression was positively associated with IPV perpetration; however, this association was not significant among couples who stipulated a sexual agreement. Length of relationship was not associated with IPV perpetration. Participants’ and partner's alcohol or drug use were not associated with IPV perpetration
Stephenson and Finneran (2016)	Minority stress and intimate partner violence among gay and bisexual men in Atlanta	USA	*N* = 1075(M)	Physical/sexual, monitoring, controlling, HIV-related and emotional IPV	IPV-GBM Scale	HIV status was not related to IPV perpetration. Homonegativity was positively associated with IPV perpetration. Internalized homonegativity was positively associated with IPV perpetration
Stephenson and Finneran (2017)	Receipt and perpetration of intimate partner violence and condomless anal intercourse among gay and bisexual men in Atlanta	USA	*N* = 1100 (M)	Physical/sexual, monitoring, controlling and emotional violence	IPV-GBM Scale	Condomless anal intercourse was associated with physical, sexual, emotional, and controlling IPV, while not with monitoring IPV
Stephenson et al., (2011a)	Intimate partner violence and sexual risk-taking among men who have sex with men in South Africa	South Africa	*N* = 521 (M)	Physical and sexual IPV	2 items developed by the authors	Age was not associated with IPV perpetration. Non-White participants were at increased risk of IPV perpetration. Less educated people were at increased risk of IPV perpetration. Number of gay friends was not associated with physical IPV perpetration. Having had partner of both sexes or only female partners, or having sex with partners other than the main partner were not related to IPV perpetration. Use of lubrification was not associated with physical IPV perpetration. Perpetrators of physical IPV were more likely to have had unprotected anal intercourse than non-perpetrators of physical IPV; no differences in unprotected anal intercourse emerged between perpetrators of sexual IPV and non-perpetrators of sexual IPV; both sexual and physical IPV were higher among participants who have had unprotected anal intercourses (UAI) compared with those who have not. Perceived stigma was not associated with IPV perpetration. Gay identity development was not related to IPV perpetration
Stephenson et al., (2011b)	Dyadic characteristics and intimate partner violence among men who have sex with men	USA	*N* = 528(M)	Emotional, physical, and sexual IPV	Four items from the Psychological Abuse scale from CTS2 to assess emotional IPV; six items developed by the authors were used to assess physical violence; three items developed by the authors were used to assess sexual coercion	Age and age differences between the partners were not associated with IPV perpetration. Ethnicity was not associated with IPV perpetration. Education was negatively associated with emotional and sexual, but not physical, IPV perpetration. Relationship satisfaction was negatively associated with psychological, but not physical or sexual, IPV perpetration. Perpetrators of emotional or physical violence showed lower levels of communal coping, couple efficacy, and couple outcome preferences; in addition, perpetrators of emotional abuse (not those who perpetrated physical or sexual abuse) had lower degree of concordance with the partner lifestyle topics. Perpetrators of sexual violence had lower communal coping scores compared with non-perpetrators, while they did not differ in couple efficacy and couple outcome preferences. Participants who reported to be HIV-positive were at increased risk of physical, but not emotional or sexual, IPV perpetration. There was a negative association between sexual IPV perpetration and perceived local stigma-couple, but not with perceived local stigma-individual. No significant associations between perceived local stigma and physical or emotional IPV perpetration were found
Stephenson et al. (2013)	Dyadic, partner, and social network influences on intimate partner violence among male–male couples	USA	*N* = 403 (M)	Physical and sexual violence	2 items developed by the authors	Having assertiveness abilities reduced the probability to perpetrate sexual coercion. Sexual victimization in the family of origin was associated with sexual IPV perpetration, while suffering physical and psychological victimization in the family of origin were not
Stults et al. (2021a)	Determinants of intimate partner violence among young men who have sex with men: the P18 cohort study	USA	*N* = 526 (M)	Physical, psychological, and sexual IPV	Three yes–no questions	Latin participants were at increased risk of IPV perpetration compared to White and Black participants. IPV perpetration was positively associated to lifetime IPV, relationship status, depression, personal gay-related stigma, and marijuana and other substance use. In contrast, SES, childhood mistreatment, impulsivity, PTSD, public gay-related stigma, and alcohol use were not associated to IPV perpetration
Stults et al., (2015a)	Intimate partner violence perpetration and victimization among YMSM: the P18 cohort study	USA	*N* = 600 (M)	Any IPV (verbal abuse, physical violence, sexual coercion)	3 items developed by the authors	Depression was positively associated with IPV perpetration; however, this relation was no longer significant when controlling for childhood maltreatment. PTSD and loneliness were positively associated with IPV perpetration at a bivariate level; however these relations were not significant in the regression model when controlling for childhood maltreatment. Impulsivity was positively associated with IPV perpetration. Involvement in LGB + support agencies was positively associated with IPV perpetration. IPV victimization was associated with IPV perpetration. Childhood maltreatment was positively associated with IPV perpetration. Personal-local stigma was positively associated with IPV perpetration, while the relation between public-gay related stigma and IPV was not significant
Stults et al. (2016)	Intimate partner violence and sex among young men who have sex with men	USA	*N* = 528(M)	Any IPV (physical, sexual and emotional)	A modified version of the Conflict Tactics Scale by Feldman et al. (2007)	Participants who reported two or more instances of anal receptive and insertive sex had higher risk of perpetrating couple violence compared with those who reported no instances of these behaviors. Unprotected sex was associated with IPV perpetration
Stults et al. (2015b)	Intimate partner violence and substance use risk among young men who have sex with men: The P18 cohort study	USA	*N* = 528(M)	Any IPV (physical, sexual and emotional)	A modified version of the conflict tactics scale by Feldman et al. (2007)	Alcohol use was not associated with IPV perpetration. Drug use was positively related to IPV perpetration
Stults et al., (2021b)	Sociodemographic differences in intimate partner violence prevalence, chronicity, and severity among young sexual and gender minorities assigned male at birth: the P18 cohort study	USA	*N* = 665 (AMAB)	Physical, sexual, psychological, any IPV, injury	CTS2	Transgender participants reported higher levels of severe injury perpetration prevalence (not minor injury, physical, psychological, and sexual IPV) than cisgender participants. Cisgender people reported higher levels of minor sexual IPV perpetration chronicity (the groups did not differ on the other forms of violence perpetrated in terms of prevalence or chronicity). Asian participants had higher levels of minor sexual IPV perpetration prevalence than White participants, while they did not differ from Latin, Black, and multiracial participants. No other differences between ethnic groups emerged on IPV perpetration prevalence. White and Black reported higher levels of minor sexual IPV perpetration chronicity than Asian participants. No other differences between ethnic groups emerged on IPV perpetration chronicity. Bisexual people reported higher levels of injury and severe sexual IPV perpetration prevalence than gay people; no other differences emerged between bisexual and gay participants in terms of IPV prevalence or chronicity. Participants who earned less than $5000 were less like to report minor psychological perpetration prevalence but more likely to report severe injury and sever sexual IPV perpetration prevalence than those who earned less. Participants who earned less than $5.000 were less likely to report minor sexual IPV perpetration chronicity than participants who earned less. No other differences emerged in IPV prevalence or chronicity between these two groups. Education was not related to IPV perpetration prevalence, while non-graduate students reported higher levels of minor psychological IPV perpetration chronicity than graduate students. No other differences emerged between these two groups
Suarez et al. (2018)	Dyadic reporting of intimate partner violence among male couples in three U.S. cities	USA	*N* = 160 same-sex couples	Physical/sexual, monitoring, controlling, HIV-related and emotional IPV	Intimate Partner Violence Among Gay and Bisexual Men (IPV-GBM) Scale	Age was negatively associated with IPV perpetration. Cohabitation was associated with increased risk of IPV perpetration. Participants’ Internalized homonegativity was positively associated with IPV perpetration, while partner’s internalized homonegativity was not
Swann et al. (2021)	Intersectional minority stress and intimate partner violence: the effects of enacted stigma on racial minority youth assigned female at birth	USA	*N* = 249 (FAB)	Severe psychological violence, severe physical violence, and sexual IPV; SGM-Specific IPV tactics	SGM-CTS2; SGM-Specific IPV Tactics Scale	Heterosexist enacted stigma was positively related to psychological and sexual IPV perpetration, but not to physical or sexual and gender minority-specific IPV. Racist enacted stigma was positively related to physical and sexual IPV perpetration. Heterosexist stigma moderated the association between racist enacted stigma and psychological IPV perpetration: this relation was significant only at low and mean levels of heterosexist stigma, while at high levels of heterosexist discrimination it was not significant. Heterosexist stigma moderated the relation between racist discrimination and sexual and gender minority-specific IPV perpetration: participants with high levels of heterosexist discrimination were at increased risk of sexual and gender minority-specific IPV perpetration than those at the mean. No other interaction effects were detected between racist and heterosexist discrimination, and IPV perpetration
Swan et al. (2021)	Discrimination and intimate partner violence victimization and perpetration among a convenience sample of LGBT individuals in Latin America	Latin America (Mexico (*n* = 92), with a minority residing in Ecuador (*n* = 2), Argentina (*n* = 1), Colombia (*n* = 1), Guatemala (*n* = 1), Paraguay (n = 1), and the Dominican Republic (n = 1))	*N* = 99, 39 (F), 51 (M), 5 (Intersex), 1 (transman), 2 (transwomen), 1 (other)	Physical, psychological, sexual IPV, injury	CTS2	All forms of IPV perpetration and victimization were significantly positively correlated; all forms of IPV were correlated to the other heterosexism subscale, but not with the other dimensions of heterosexism (harassment/rejection; heterosexism at work/school)
Taylor and Neppl (2020)	Intimate partner psychological violence among GLBTQ college students: The role of harsh parenting, interparental conflict, and microaggressions	USA	*N* = 379, 228 (F), 106 (M), 45 (gender minority)	Psychological violence	CTS2	Experiencing microaggressions was positively associated with IPV perpetration, and this relation was moderated by sexual orientation (i.e., having a bisexual orientation increased the strength of the association between microaggressions and IPV perpetration)
Telesco (2003)	Sex role identity and jealousy as correlates of abusive behavior in lesbian relationships	USA	*N* = 105(F)	Physical and psychological abuse + a total score	The 30 item abusive behavior inventory (ABI)	Dependence was not related to IPV perpetration. Jealousy was positively associated with IPV perpetration. Perceived power imbalances were not associated with IPV perpetration. Gender expression was not associated with IPV perpetration
Tognasso et al. (2022)	Romantic attachment, internalized homonegativity and same-sex intimate partner violence perpetration among lesbian women in Italy	Italy	*N* = 325, 311(F), 2 (transgender women), 12 (other)	Physical, psychological, sexual, and any IPV	CTS2S	Attachment avoidance was positively related to psychological, physical, and any IPV, but not to sexual IPV. The association between Attachment avoidance, and psychological and any IPV was partially mediated by internalized homonegativity. Attachment anxiety was positively related to psychological and any IPV perpetration, but not to physical and sexual IPV. These associations were partially mediated by internalized homonegativity
Toro-Alfonso and Rodríguez-Madera (2004)	Sexual coercion in a sample of Puerto Rican gay males	Puerto Rico	*N* = 302 (M)	Sexual coercion	A questionnaire developed by the authors	Having assertiveness abilities reduced the probability to perpetrate sexual coercion. Sexual victimization in the family of origin was associated with sexual IPV perpetration, while suffering physical and psychological victimization in the family of origin were not. Addictive behaviors were positively associated with IPV perpetration
Turell et al. (2018)	Disproportionately high: an exploration of intimate partner violence prevalence rates for bisexual people	US	*N* = 439, 184 (M), 206 (F), 5 (transwomen), 4 (transmen), 35 (genderqueer/fluid), 5 (Undecided)	Any IPV (physical and psychological abuse)	ABI	Participants' age and age differences between the partners were not associated with IPV perpetration; partner's age was negatively associated with IPV perpetration. Gender identity was not associated with IPV perpetration. Having a bisexual partner was associated with IPV perpetration. Black/African American and indigenous participants were at increased risk of IPV perpetration. Length of relationship was not associated with IPV perpetration. Having a child was not associated with IPV perpetration. Being in an open relationship and infidelity were both associated with abuse perpetration. Bisexual participants involved in bisexual local or online community were at increased risk of IPV perpetration than those not involved in bisexual communities; however, in the path analysis, involvement in bisexual communities was not directly associated with IPV perpetration. Bi-negativity was positively associated with IPV perpetration
Ummak et al. (2021)	Untangling the relationship between internalized heterosexism and psychological intimate partner violence perpetration: a comparative study of lesbians and bisexual women in Turkey and Denmark	Turkey and Denmark	*N* = 449, 418 (F), 10 (transgender), 12 (complex)	Psychological IPV	MMEA Scale	Turkish participants were more likely to report all forms of psychological IPV perpetration (restrictive engulfment; denigration; hostile withdrawal; dominance/intimidation) than Danish participants. Bisexual participants were more likely to report all forms of psychological IPV perpetration, except for dominance/intimidation, than Lesbian participants. Internalized heterosexism was positively related to each form of IPV perpetration. Sexual orientation moderated only the relation between internalized heterosexism and dominance/intimidation: among bisexual this relation was not significant. This interactional effect was found both among Turkish and Danish participants. No other moderating effects of sexual orientation or country were detected
Waterman et al. (1989)	Sexual coercion in gay and lesbian relationships: predictors and implications for support services	USA	*N* = 70, 36 (F), 34 (M)	Forced sex; Physical violence	1 item developed by authors assessing forced sex perpetration; CTS to assess physical violence	No gender differences in IPV perpetration were found. Physical IPV victimization was associated with physical IPV perpetration, while the association between sexual IPV victimization and perpetration was significant only among men
Wei et al. (2020a)	Multilevel factors associated with perpetration of five types of intimate partner violence among men who have sex with men in China: an ecological model-informed study	China	*N* = 578 (M)	Physical IPV; sexual IPV; monitoring IPV; controlling IPV; emotional IPV; a total score (any IPV)	IPV-GBM Scale	Bisexual people were at increased risk of IPV perpetration compared to homosexual people. Self-esteem was negatively associated with IPV perpetration; Self-efficacy was negatively associated with emotional IPV perpetration. Perceived instrumental support by family, friends and colleagues was negatively associated with IPV perpetration. Involvement in social activities within the LGB community was positively associated with IPV perpetration. Emotional, controlling, monitoring, sexual, and physical IPV perpetration were all correlated to each other. Drug use during sex was associated with IPV perpetration. An age of 18 or older at sexual debut was positively associated with controlling behaviors and negatively related to emotional IPV. Number of sexual partners was positively associated with IPV perpetration. Perceived stigma was positively associated with IPV perpetration
Wei et al. (2020b)	Effects of emotion regulation and perpetrator- victim roles in intimate partner violence on mental health problems among men who have sex with men in China	China	*N* = 578 (M)	Physical, sexual, monitoring, controlling, emotional, any IPV	Five items derived from the IPV-GBM Scale	Age, ethnicity, education level, marital status, job, and sexual orientation were not related to IPV perpetration. Age of first homosexual intercourse of 18 or older was negatively associated with physical and psychological perpetration, but not with sexual, monitoring, or controlling IPV. Higher self-esteem was negatively associated only with sexual violence perpetration. Being ever engaged in transactional sex was positively associated only with perpetration of monitoring IPV. Drug use was not related to any form of IPV perpetration. Physical and monitoring, physical and emotional, sexual, and controlling, sexual and emotional, and monitoring and emotional IPV perpetration were positively associated with each other. No other association between the forms of perpetrated IPV emerged. Any IPV perpetration and any IPV victimization were associated with each other
Wei et al. (2021)	Prevalence of intimate partner violence and associated factors among men who have sex with men in China	China	*N* = 431 (M)	Physical, sexual, monitoring, controlling, and emotional IPV	IPV-GBM Scale	Monitoring and any IPV perpetration (not physical, emotional, sexual, and controlling IPV) were positively associated to suicidality. Monitoring, controlling, emotional, and any IPV perpetration (not physical and sexual IPV) were negatively related to general mental health. Emotional and monitoring IPV (not physical, emotional, sexual, and controlling IPV) were positively related to depression
Whitton et al. (2019)	Intimate partner violence experiences of sexual and gender minority adolescents and young adults assigned female at birth	USA	*N* = 352 (F)	Minor psychological IPV, Severe psychological IPV, Minor physical IPV, Severe physical IPV, Injury, Sexual IPV, Coercive control, SGM-specific IPV, Cyber abuse + a total score	The SGM Conflict Tactics Scale 2 (SGM-CTS2); The Coercive Control Scale; the SGM-Specific IPV Tactics Scale; The Cyber Abuse Scale	Age was not associated with IPV perpetration. Participants’ gender identity was not associated with IPV perpetration; participants with a gender minority partner were at increased risk of IPV perpetration than participants with a cisgender partner. Sexual orientation was not associated with IPV perpetration. Black and Latin participants were at increased risk of IPV perpetration
Whitton et al. (2021)	Exploring mechanisms of racial disparities in intimate partner violence among sexual and gender minorities assigned female at birth	USA	*N* = 308 (AFAB SGM)	Minor psychological, severe psychological, physical, and sexual IPV	SGM-CTS2	Black participants were more likely to report physical, psychological, and sexual IPV perpetration than White participants; Latinx participants were more likely to report more severe psychological, and physical, and sexual IPV perpetration than White participants. No other differences emerged between Black, Latin, and White participants on minor or severe psychological, physical, or sexual IPV perpetration. Child abuse experiences, witnessing violence between parents, and racial discrimination were related to each form of IPV. Economic stress and social support were related to each form of violence except for minor psychological perpetration. Sexual and Gender Minority victimization was positively related only to minor psychological IPV perpetration, while internalized sexual stigma was not related to IPV perpetration. Identifying as Black or Latinx (vs. White) had an indirect effect on severe psychological perpetration via racial discrimination, identifying as Black or Latinx (vs. White) was indirectly associated with minor psychological perpetration through child abuse. Direct effects of race were nonsignificant in these models, except for Black identity in the prediction of severe psychological perpetration and physical perpetration. No other indirect effects of child abuse experiences, witnessing violence between parents, economical stress, racial discrimination, or social support in the association between ethnicity and IPV perpetration
Wong et al. (2010)	Harassment, discrimination, violence, and illicit drug use among young men who have sex with men	USA	*N* = 526(M)	Physical violence	Adaptation of a scale developed by Smith et al. (1995) to measure intimate partner violence among battered women, including 3 items asking about physical violence perpetration	Caucasian participants were more likely to report physical and emotional, but not sexual, IPV perpetration than African American participants. Drug use was related to IPV perpetration
Wu et al. (2015)	The association between substance use and intimate partner violence within Black male same-sex relationships	USA	*N* = 74 (M)	Psychological, physical, sexual and injurious IPV	CTS2	Alcohol use was positively associated with IPV perpetration. Methamphetamine use was associated with IPV perpetration, while marijuana, powered or rock/crack cocaine, or heroin use were not
Zavala (2017)	A multi-theoretical framework to explain same-sex intimate partner violence perpetration and victimization: a test of social learning, strain, and self-control	USA	*N* = 665, 195 (M), 470 (F)	Any IPV	CTS2	Age was positively associated with IPV perpetration. Non-white participants were at increased risk of IPV perpetration. Depression was positively associated with IPV perpetration. Self-control was negatively associated with IPV perpetration. Social support was not related to IPV perpetration. Anti-gay violence was positively associate with IPV perpetration. Perceived stigma was not associated with IPV perpetration. Internalized homonegativity was positively associated with IPV perpetration
Zhu et al. (2021)	Moderating effect of self-efficacy on the association of intimate partner violence with risky sexual behaviors among men who have sex with men in China	China	*N* = 578 (M)	Physical, sexual, monitoring, controlling, emotional, and any IPV	IPV-GBM	Inconsistent condom use with regular partners was positively associated to monitoring and any IPV perpetration, while not to physical, emotional, sexual, and controlling IPV perpetration. Inconsistent condom use with casual partners and multiple regular partners was positively related only to sexual IPV perpetration. Having multiple casual sexual partners was only related to emotional, monitoring, and any IPV perpetration. Self-efficacy moderated the relation between multiple casual sexual partners and emotional IPV perpetration: at high levels of self-efficacy the relation between multiple casual sexual partners and emotional IPV perpetration was no longer significant. No other moderating effect of self-efficacy on the association between risky sexual behaviors and IPV perpetration were found

#### Sociodemographic Factors

##### Gender Differences

No gender differences in perpetration of IPV were found in eight studies (Chong et al., 2013; Craft et al., 2008; Fontanesi et al., 2020; Gabbay & Lafontaine, 2017b; Longares et al., 2018b; Pistella et al., 2022; Reuter et al., 2015; Waterman et al., 1989). In contrast, in the study by Derlega et al., (2011), the results showed that men who were rejected after the breakup of a relationship engaged in more pursuit behaviors (i.e., invasive and annoying, but not necessarily threatening behaviors) than women did, though gender differences were not found considering aggressive behaviors (i.e., invasive and threatening behaviors).

**Age** Suarez et al. (2018) identified a negative association between participants’ age and IPV perpetration. Similarly, Turell et al. (2018) identified a negative relation between partner’s age and IPV perpetration. In contrast, two studies showed a positive association between age and couple violence perpetration (Messinger et al., 2021; Zavala, 2017). In Finneran et al. (2012), participants aged between 25 and 40 years old were at increased risk of perpetration of sexual IPV only in the USA, while no significant association between age and IPV perpetration was found in Canada, Australia, UK, Brazil, and South Africa. The remaining articles that assessed the association between IPV perpetration and participants’ age (Balsam & Szymanski, 2005; Bartholomew et al., 2008a, b; Chong et al., 2013; Finneran & Stephenson, 2014; Pistella et al., 2022; Stephenson et al., 2011a; Stephenson et al., 2011a, b; Turell et al., 2018; Whitton et al., 2019; Wei et al., 2021) or age differences between the partners (Stephenson et al., 2011a, b; Turell et al., 2018) did not find significant results.

**Gender Identity** No differences between participants’ gender identities (i.e., cisgender vs. gender minorities) in the perpetration of IPV were found in two studies (Turell et al., 2018; Whitton et al., 2019). In contrast, in Stults et al., (2021a, b), transgender participants reported a higher injury perpetration prevalence compared to cisgender participants, while this relation was not significant when considering physical, psychological, and sexual IPV prevalence. Minor sexual IPV chronicity was instead more common among cisgender participants than transgender ones in this study (Stults et al., 2021a, b). No differences emerged in relation to the other assessed forms of IPV. In the study by Whitton et al. (2019), compared to participants with cisgender female partners, those with gender minority partners were at increased risk for perpetrating coercive control and abusive tactics specific of sexual and gender minorities.

**Sexual Orientation** Bisexual people were at increased risk of IPV perpetration compared to homosexual people in four studies (Reuter et al., 2015; Stults et al., 2021a, b; Ummak et al., 2021; Wei et al., 2020a, b). In the study by Reuter et al. (2015), the linear regression model further showed that sexual orientation was only related to severe teen dating violence (TDV; i.e., a combination of physical and sexual IPV) perpetration, while it was not related to TDV when any TDV score was considered as a dependent variable. Finally, Turell et al. (2018) found an association between having a bisexual partner and IPV perpetration. In contrast, sexual orientation was not associated with IPV perpetration in five studies (Chong et al., 2013; Finneran & Stephenson, 2014; Miltz et al., 2019; Wei et al., 2021; Whitton et al., 2019).

**Ethnicity** Non-White participants were found to be at increased risk of IPV perpetration in three studies (Messinger et al., 2021; Stephenson et al., 2011a; Zavala, 2017), and Black/African American and indigenous participants reported higher rates of IPV perpetration in the study by Turell et al. (2018). Similarly, Black and Latin participants were at increased risk of IPV perpetration compared to White participants in the study by Whitton et al. (2019), while in a more recent study by Whitton et al. (2021), only Black, and not Latin participants, were at increased risk of IPV perpetration compared to White participants. In contrast, Latin participants were at increased risk of IPV perpetration compared with Black and White participants in the study by Stults et al. (2021a). In a more recent study by Stults et al., (2021a, b), Asian participants had higher levels of minor sexual IPV perpetration prevalence compared to White participants (and not compared to Latin, Black, or multi-ethnic participants), while this relation was not significant in relation to physical, psychological, and severe sexual IPV perpetration prevalence. There were no differences in IPV perpetration prevalence between White, Latin, and Black participants. On the other hand, IPV perpetration chronicity was more common among White and Black participants than among Asian participants in this study (Stults et al., b). Caucasian participants were more likely to report physical and emotional, but not sexual, IPV perpetration than African American participants in one study (Wong et al., 2010). Turkish participants were at increased risk of IPV perpetration compared with Danish participants in Ummak et al. (2021). Six studies did not identify a significant association between ethnicity and perpetration of IPV (Finneran & Stephenson, 2014; Finneran et al., 2012; Fortunata & Kohn, 2003; Miltz et al., 2019; Stephenson et al., 2011a, b; Wei et al., 2021).

**Education** Less educated people were at increased risk of IPV perpetration in 9 studies (Balsam & Szymanski, 2005; Bartholomew et al., 2008a, b; Chong et al., 2013; Finneran & Stephenson, 2014; Milletich et al., 2014; Miltz et al., 2019; Stephenson et al., 2011a; Stephenson et al., 2011a, b; Stults et al., 2021a, b). However, in the study by Bartholomew et al., (2008a, b), the association between physical and psychological IPV and education was not significant when controlling for the effect of IPV victimization (i.e., bidirectionality of abuse). In addition, the negative association between education and lifetime physical and sexual IPV perpetration that emerged in the study by Balsam and Szymanski (2005) was not significant when recent IPV was considered as the dependent variable. Finneran and Stephenson (2014) found that only physical IPV perpetration was negatively associated with education, while sexual IPV was not. In contrast, less educated people were at increased risk of perpetrating sexual and emotional, but not physical, abuse in the study by Stephenson et al., (2011a, b). In the study by Finneran et al. (2012), participants with more than 12 years of education were at increased risk of IPV perpetration only in Canada, while this relation was not significant in the USA, Australia, UK, Brazil, or South Africa. Education was not related to IPV perpetration in one study (Wei et al., 2021).

**Income** Income was negatively associated with physical, but not sexual, IPV perpetration in one study (Chong et al., 2013). Similarly, in the study by Bartholomew et al., (2008a, b), income was negatively associated with physical, but not emotional, IPV perpetration. However, this relation was no longer significant when controlling for IPV victimization. Fortunata and Kohn (2003) found that batterers’ partners had lower income than non-batterers’ partners, while income was not associated with IPV perpetration in Balsam and Szymanski (2005). Finally, income was negatively related to severe injury and severe sexual IPV perpetration prevalence, and positively related to minor psychological IPV prevalence and minor sexual IPV perpetration chronicity in Stults et al., (2021a, b). Income was not associated to minor injury, minor sexual, severe psychological, and physical IPV perpetration prevalence, and to chronicity of injury, physical, psychological, and severe sexual IPV perpetration in this study (Stults et al., 2021a, b).

**Employment** None of the studies that assessed the association between employment and IPV perpetration highlighted significant results (Finneran & Stephenson, 2014; Fortunata & Kohn, 2003; Miltz et al., 2019; Wei et al., 2021).

#### Psychological Factors

**Mental Health (General Mental Health; Emotion Regulation; Depression, Anxiety, and PTSD; Loneliness; Stress; Suicidality; COVID-19)** In Wei et al., (2020a, b), general mental health was associated to any, emotional, controlling, and monitoring IPV, while these results were not supported in relation to physical and sexual IPV. Cognitive reappraisal was associated with IPV perpetration in one study (Wei et al., 2020a, b), while expressive suppression was not.

Depression was positively associated with IPV perpetration among sexual minority people in six studies (Miltz et al., 2019; Sharma et al., 2021; Stults et al., 2015a, 2021a; Wei et al., 2020a, b; Zavala, 2017). However, in the research by Sharma et al. (2021), the association was not significant among couples who stipulated a sexual agreement. In the study by Stults et al. (2015a), this relation was no longer significant when controlling for childhood maltreatment. Bacchus et al. (2017) did not find a significant association between depression and IPV perpetration.

In addition, a marginal association between symptoms of mild anxiety disorder and any negative behaviors in the past 12 months (i.e., any abusive behaviors, which include physical abuse, frightening, forcing sex, and controlling behaviors perpetrated in the past 12 months) was found in Bacchus et al. (2017). Symptoms of mild anxiety disorder were not associated with physical abuse, frightening, forcing sex, or controlling behaviors in this study (Bacchus et al., 2017).

PTSD was positively associated with IPV perpetration at a bivariate level in one study (Stults et al., 2015a); however, this relation was not significant in the regression model when controlling for childhood maltreatment. Also, Stults et al. (2021a) did not find a significant association between PTSD and IPV perpetration.

In the study by McKenry et al. (2006), non-perpetrating females reported higher psychological adjustment compared with non-perpetrating males, and male and female perpetrators. A positive association between psychological maladjustment and psychological IPV perpetration was confirmed in Pepper and Sand (2015), although psychological maladjustment was not related with sexual or physical IPV in this study.

Stults et al. (2015a) identified a positive association between IPV perpetration and loneliness at a bivariate level. However, this relation was no longer significant in the regression analysis when controlling for childhood maltreatment. In the study by McKenry et al. (2006), IPV perpetrators experienced more family stress than non-perpetrators, and the relation between perceived stress and IPV perpetration was fully mediated by insecure attachment in the study by Craft et al. (2008). Similarly, in Whitton et al. (2021) economic stress was associated to physical, sexual, and severe psychological IPV perpetration, while this association was not significant when considering minor psychological IPV. Any IPV perpetration and controlling behavior were positively related to suicidality in one study (Wei et al., 2020a, b). These results were not supported in relation to physical, emotional, controlling, and sexual IPV perpetration.

Finally, Pistella et al. (2022) did not highlight an association between COVID-19 psychosocial impact and IPV perpetration.

**Personality Traits** An association between at least some personality traits and IPV perpetration was found in seven studies (Chong et al., 2013; Fortunata & Kohn, 2003; Landolt & Dutton, 1997; Pepper & Sand, 2015; Reuter et al., 2015; Stults et al., 2015a; Zavala, 2017). Specifically, the results found by Landolt and Dutton (1997) showed that both actor’s and partner’s abusive personality (i.e., constituted by borderline personality organization [BPO], anger, fearful attachment, preoccupied attachment, maternal rejection and paternal rejection) was associated with physical and psychological IPV perpetration. More specifically, each constituent of the abusive personality of both the actor and the partner were associated with psychological IPV perpetration. For physical IPV perpetration both actor and partner effects were significant for BPO, fearful, and preoccupied attachment, while neither actor nor partner effects were significant for anger and paternal rejection, and only actor effects were significant for maternal rejection (Landolt & Dutton, 1997).

In the study by Fortunata and Kohn (2003), a relation between personality traits and IPV perpetration was confirmed: batterers had higher scores on the aggressive (sadistic), antisocial, avoidant, passive-aggressive, self-defeating, borderline, paranoid, and schizotypal personality scale scores and higher alcohol-dependent, drug-dependent, bipolar (manic syndrome), and delusional clinical syndrome scale scores. However, no significant differences between batterers and non-batterers emerged in the scores on compulsive, dependent, depressive, histrionic, narcissistic, schizoid, anxiety, dysthymia, PTSD, somatoform, major depression, and thought disorders scales. When controlling for desirability and debasement, group differences for the avoidant, bipolar (manic syndrome), dependent, passive-aggressive, schizoid, schizotypal, and self-defeating personality were no longer significant (Fortunata & Kohn, 2003).

In addition, hostility was positively associated with perpetration of IPV in two studies (Pepper & Sand, 2015; Reuter et al., 2015), as was impulsivity in the study by Stults et al. (2015a), even after controlling for the effect of childhood maltreatment. However, impulsivity was not associated with IPV perpetration in Stults et al. (2021a). Self-control (Zavala, 2017) and anger management (Chong et al., 2013) were found to be negatively associated to IPV perpetration. However, in the study by Chong et al. (2013), the relation between physical IPV perpetration and anger management was fully mediated by psychological IPV perpetration. Emotional instability was positively related with physical and psychological IPV perpetration, but not with sexual IPV, in the study by Pepper and Sand (2015). Furthermore, these authors found a significant association between negative worldview and psychological IPV perpetration, while this relation was not significant when physical or sexual IPV were considered as dependent variables (Pepper & Sand, 2015). Emotional unresponsiveness was not associated to IPV perpetration (Pepper & Sand, 2015).

**Self-Esteem and Self-Efficacy** Self-esteem was negatively associated to IPV perpetration in four studies (Causby et al., 1995, McKenry et al., 2006; Wei et al., 2020a, b; Wei et al., 2021). However, negative self-esteem was not associated to IPV perpetration in Pepper and Sand (2015).

Self-efficacy was negatively associated to emotional IPV perpetration in the study by Wei et al., (2020a, b). In contrast, two studies did not find significant association between physical and sexual IPV perpetration and self-efficacy or self-adequacy (Chong et al., 2013; Pepper & Sand, 2015).

**Attachment** In the study by Longares et al. (2018a), the results highlight a significant association between insecure adult attachment and psychological IPV. Outness moderated this relation: at low levels of overall outness, the relationship between insecure attachment and psychological IPV was not significant. Similarly, at low and high levels of outness to religion, this association was not significant. Outness to the family did not moderate the association between insecure attachment and psychological IPV (Longares et al., 2018a). According with these findings, in the study by McKenry et al. (2006), perpetrators had a less secure attachment style than non-perpetrators. Fontanesi et al. (2020) identified a negative association between confidence (i.e., a dimension that represents a secure attachment style) and commitment defection and manipulation (i.e., two of the three dimensions of sexual abuse in this study), and, surprisingly, a positive association between confidence and emotional abuse. No significant relationship between confidence and coercion of resources and violence (i.e., the third dimension of sexual abuse) was detected (Fontanesi et al., 2020).

In addition, four studies found a significant association between attachment anxiety and IPV perpetration (Bartholomew et al., 2008a, b; Derlega et al., 2011; Gabbay & Lafontaine, 2017a; Tognasso et al., 2022). The association between attachment anxiety and sexual IPV perpetration was fully mediated by dyadic trust and sexual intimacy in a serial mediation model in the study by Gabbay and Lafontaine (2017a). In the study by Derlega et al. (2011), only the relation between attachment anxiety and pursuit behaviors was significant, while the relationship between attachment anxiety and aggressive behaviors was not. In Tognasso et al. (2022), attachment anxiety was related to any and psychological IPV perpetration, and this relation was partially mediated by internalized homonegativity. Attachment anxiety was not associated to physical and sexual IPV in this study (Tognasso et al., 2022). Furthermore, only attachment anxiety assessed through interview, and not self-reported anxious attachment, was still associated to physical and psychological IPV perpetration in the study by Bartholomew et al., (2008a, b) when controlling for IPV victimization (i.e., bidirectionality of abuse). Gabbay and Lafontaine (2017b) did not identify a significant relation between self-reported attachment anxiety and physical or psychological IPV perpetration.

Four studies highlighted a significant association between attachment avoidance and IPV perpetration (Bartholomew et al., 2008a, b; Gabbay & Lafontaine, 2017a, b; Tognasso et al., 2022). However, in the study by Bartholomew et al., (2008a, b), only attachment avoidance assessed through interview was associated to physical and psychological IPV perpetration (even after controlling for IPV victimization), while self-reported avoidance was not. Attachment avoidance was only associated to physical, and not psychological IPV perpetration in the study by Gabbay and Lafontaine (b), and this relation was no longer significant when controlling for receipt of violence. In Tognasso et al (2022), the association between attachment avoidance and sexual IPV was not significant. However, they highlighted a direct relation between attachment avoidance and physical IPV, and a positive association between attachment avoidance, and any and psychological IPV, partially mediated by internalized homonegativity. Furthermore, the relation between attachment avoidance and sexual IPV perpetration was partially mediated by dyadic trust and sexual intimacy in a serial mediation model in the study by Gabbay and Lafontaine (2017a). Derlega et al. (2011) did not find any association between attachment avoidance and pursuit or aggressive behaviors. In addition, discomfort with closeness, need for approval, and preoccupation with relationships were all related with some dimension of sexual coercion or emotional abuse (see Table 2 for more details) in the study by Fontanesi et al. (2020). The relationship being secondary was not associated to emotional abuse or sexual coercion in this study (Fontanesi et al., 2020).

The proximity dimension of caregiving (and not sensitivity, compulsive caregiving, and controlling caregiving) was negatively associated to physical and psychological IPV perpetration in the research by Gabbay and Lafontaine (2017b), although this relation was not significant when controlling for receipt of violence. In addition, these authors identified a significant association between psychological IPV perpetration and both hyperactivation of the attachment and caregiving systems and deactivation of the attachment and caregiving systems, even in the presence of each other. Regarding physical IPV perpetration, only hyperactivation was still associated to physical couple violence when controlling for the effect of deactivation strategies. None of these findings were significant when receipt of violence was controlled for (Gabbay & Lafontaine, 2017b).

#### Relational Factors

**Couple-Level Demographic Factors** Cohabitation was correlated with increased risk of IPV perpetration in the study by Suarez et al. (2018), while cohabitation with a same-sex partner was not associated to physical or psychological IPV perpetration in Chong et al. (2013). Length of relationship was not associated to IPV perpetration in four studies (Chong et al., 2013; Pistella et al., 2022; Sharma et al., 2021; Turell et al., 2018), and neither was having a child in the studies of Fortunata and Kohn (2003) and Turell et al. (2018). Relationship status (i.e., single, married, having boyfriend or other) did not predict IPV perpetration as well (Wei et al., 2021).

**Open Relationships, Monogamy, and Infidelity** Being in an open relationship and infidelity were both associated to abuse perpetration in one study (Turell et al., 2018). In contrast, batterers and non-batterers did not differ regarding monogamous relationships in Fortunata and Kohn (2003).

**Couple Dynamics** Three studies found a negative association between dyadic adjustment or relationship satisfaction, and IPV perpetration (Balsam & Szymanski, 2005; Li et al., 2019; Stephenson et al., 2011a, b). However, in the studies by Li et al. (2019) and Stephenson et al., (2011a, b), only psychological IPV was associated to relationship satisfaction, while physical, and physical or sexual IPV respectively were not. In addition, in two studies relationship satisfaction was not associated to IPV perpetration (Derlega et al., 2011; McKenry et al., 2006).

Sexual satisfaction was negatively associated to IPV perpetration in one study (Pistella et al., 2022). In the study by Poorman and Seelau (2001), perpetrators had lower expressed and wanted inclusion, and expressed and wanted affection compared with non-perpetrators. However, expressed and wanted control did not differ between perpetrators and non-perpetrators, and there were no differences between the groups in the differences between expressed and wanted inclusion, expressed and wanted affection or expressed, and wanted control (Poorman & Seelau, 2001). Perpetrators of emotional or physical violence showed lower levels of communal coping, couple efficacy, and couple outcome preferences in the study by Stephenson et al., (2011a, b). In addition, perpetrators of emotional abuse (not those who perpetrated physical or sexual abuse) had lower degree of concordance with the partner lifestyle topics. Perpetrators of sexual violence had lower communal coping scores compared with non-perpetrators, while they did not differ in couple efficacy and couple outcome preferences (Stephenson et al., 2011a, b). Furthermore, higher scores in investment size (i.e., personal investment in the relationship), not in poor quality of alternatives or commitment in relationships, were related with unwanted pursuit (and not with aggressive behaviors) in the study conducted by Derlega et al. (2011).

Finally, while dependence was not related with IPV perpetration in three studies (McKenry et al., 2006; Pepper & Sand, 2015; Telesco, 2003), jealousy (Telesco, 2003) and fusion/intrusiveness (i.e., enmeshment in one’s couple relationship; Causby et al., 1995; Mason et al., 2016; Milletich et al., 2014) were found to be both positively associated to IPV perpetration. In the study by Causby et al. (1995), while share fusion was associated to physical aggression, physical/more severe violence, and psychological violence, time fusion was only associated to physical aggression and psychological violence.

**Power Dynamics** In the study by Landolt and Dutton (1997), perpetration of psychological IPV by abusers was higher when victims perceived to be in a divided-power couple compared to when victims perceived to be in an egalitarian couple. No other differences regarding psychological IPV perpetration emerged when comparing victims’ perception of being in a divided-power, egalitarian, or self-dominant couple. Perceived power differentials or power imbalances were not associated to IPV perpetration in two studies (McKenry et al., 2006; Telesco, 2003). In addition, in the study by Landolt and Dutton (1997), couples that disagreed in their perception of relationship power dynamics (i.e., non-congruent couples) did not differ from congruent couples in their levels of IPV perpetration. Finally, dominance was positively associated to IPV perpetration in the study by Chong et al. (2013). However, this relation was no longer significant when controlling for demographic variables. Milletich et al. (2014) did not identify a significant relation between dominance/accommodation and IPV perpetration. Nevertheless, these authors found an indirect influence of accommodation on IPV perpetration through fusion: accommodation was positively related with fusion, which in turn was positively associated to IPV perpetration (Milletich et al., 2014).

**Conflict, Conflict Resolution Skills, and Communication** Relationship conflict was positively associated to physical and psychological IPV in two studies (Chong et al., 2013; Pistella et al., 2022), while having assertiveness abilities reduced the probability to perpetrate sexual coercion in one study (Toro-Alfonso & Rodríguez-Madera, 2004). Finally, Krahé et al. (2000) found a significant association between token resistance (i.e., one of the two dimensions of ambiguous communication during sexual encounters, which describes the tendency to refuse sex when actually it is what one desires) and sexual violence, while the relation between sexual violence and compliance (i.e., the second dimension of ambiguous communication during sexual encounters, which describes the tendency of having sex with someone when one does not want to) was not significant.

#### Social- and Community-Level Factors

**Characteristics of the Social Network and Social Support** Perceived instrumental support by family, friends, and colleagues was negatively associated to IPV perpetration in two studies (Wei et al., 2020a, 2020b; Whitton et al., 2021). However, in Whitton et al. (2021), this relation was significant only when considering physical, sexual, and severe psychological IPV perpetration, while it was not supported when considering minor psychological IPV. In contrast, social support was not related with IPV perpetration in three studies (Edwards et al., 2021; Reuter et al., 2015; Zavala, 2017). Similarly, the number of gay friends was not associated to physical IPV perpetration in Stephenson et al. (2011a), while being involved in a male network composed by perpetrators of violence was positively associated to dating or sexual violence only among lesbian women and not among gay men in the study by Jones and Raghavan (2012).

**Involvement in LGB Communities and Support Agencies** Involvement in social activities within the LGB community (Wei et al., 2020a, b) and involvement in LGB + support agencies (Stults et al., 2015a) were both positively associated to IPV perpetration. Furthermore, in the study by Turell et al. (2018), the analysis of variance showed that bisexual participants involved in local or online bisexual communities were at increased risk of IPV perpetration than those not involved in bisexual communities. However, in the path analysis, involvement in bisexual communities was not associated to IPV perpetration (Turell et al., 2018). Pistella et al. (2022) did not find a significant association between LGB community involvement and IPV perpetration.

**Ethnic Discrimination** Two studies identified a positive association between ethnic discrimination and IPV perpetration (Swann et al., 2021; Whitton et al., 2021).

**Religiosity** Pistella et al. (2022) did not find a significant association between religiosity and IPV perpetration.

#### Feminine and Masculine Gender Expression and Sexism

Masculinity was positively associated to IPV perpetration in three studies (Jacobson et al., 2015; McKenry et al., 2006; Oringher & Samuelson, 2011). However, in Oringher and Samuelson (2011), only some dimensions of masculinity were associated to physical IPV perpetration: suppression of vulnerability and aggressiveness were both positively related to physical IPV perpetration, while avoidance of dependency on other was negatively related with physical IPV perpetration. In contrast, the association between self-destructive achievement and dominance, and physical IPV perpetration was not significant, and no dimensions of masculinity were associated with sexual IPV perpetration (Oringher & Samuelson, 2011). In the studies by Telesco (2003) and Balsam and Szymanski (2005), the relationship between gender expression and IPV perpetration was not significant.

In the study by Li and Zheng (2021), both benevolent or hostile sexism toward women and hostile sexism toward men were positively associated to cold violence perpetration. These associations were not significant when considering any IPV and controlling violence perpetration. Benevolent sexism toward men was not associated to any IPV, cold violence, or controlling behaviors perpetration in this study (Li & Zheng, 2021).

#### Intimate Partner Violence

In 17 studies, IPV victimization was positively associated to IPV perpetration (Bartholomew et al., 2008a, b; Edwards et al., 2021; Gabbay & Lafontaine, 2017b; Lewis et al., 2017; Li & Zheng, 2021; Longares et al., 2018b; Miltz et al., 2019; Oringher & Samuelson, 2011; Pepper & Sand, 2015; Pistella et al., 2022; Stults et al., 2015a, b, 2021a; Swan et al., 2021; Waterman et al., 1989; Wei et al., 2021). However, while Pepper and Sand (2015) found a significant relation between physical IPV victimization and perpetration, and between psychological IPV victimization and perpetration, they did not find a significant association between sexual IPV victimization and perpetration. In Lewis et al. (2017), while physical violence perpetration and victimization were each other associated, only the association between psychological IPV perpetration and psychological IPV victimization was significant, but the opposite directional path was not. In addition, in the study by Waterman et al. (1989), the association between sexual IPV victimization and perpetration was significant only among sexual minority men, and not among sexual minority women. The association between physical IPV victimization and physical IPV perpetration was significant for both genders in this study (Waterman et al., 1989).

In six studies (Bartholomew et al., 2008a, b; Chong et al., 2013; Finneran & Stephenson, 2014; Wei et al., 2020a, b, 2021), different forms of IPV perpetrated by participants were all significantly associated to each other (see Table 2 for more details). However, Finneran and Stephenson (2014) found a significant association only between sexual and psychological IPV perpetration, and between psychological and physical IPV perpetration, while the association between sexual and physical IPV perpetration was no longer significant in the logistic model when controlling for the effect of other variables.

#### Family of Origin-Related Factors

**Witnessing Violence in the Family of Origin** Witnessing IPV between parents (Messinger et al., 2021; Schilit et al., 1991; Whitton et al., 2021) or siblings (Messinger et al., 2021) was positively associated to IPV perpetration in three studies. In Craft and Serovich (2005), only witnessing violence from mother-to-father was associated to sexual coercion perpetration, while witnessing violence from father-to-mother was not. No significant associations were found between witnessing violence (both from mother-to-father and from father-to-mother) and psychological IPV, physical assault, or physical injury perpetration in this study (Craft & Serovich, 2005). Similarly, five other studies did not identify a significant association between these variables (Bartholomew et al., 2008a, b; McKenry et al., 2006; Milletich et al., 2014; Reuter et al., 2015).

**Childhood Maltreatment and Harsh Parenting** Childhood maltreatment was positively associated to IPV perpetration in six studies (Fortunata & Kohn, 2003; Messinger et al., 2021; Schilit et al., 1991; Stults et al., 2015a; Toro-Alfonso & Rodríguez-Madera, 2004; Whitton et al., 2021). However, only sexual victimization in the family of origin was associated to sexual IPV perpetration in the study by Toro-Alfonso and Rodríguez-Madera (2004), while suffering physical and psychological victimization in the family of origin were not. In addition, in the study by Bartholomew et al., (2008a, b), a positive association was found between IPV perpetration and mother-to-teen violence, while the relation between father-to-teen violence and IPV perpetration was not significant. The association between mother-to-teen violence was no longer significant when controlling for IPV victimization. Similarly, in other studies, childhood maltreatment (Chong et al., 2013; McKenry et al., 2006; Milletich et al., 2014; Stults et al., 2021a) or harsh parenting (Taylor & Neppl, 2020) were not related with IPV perpetration.

There were no differences between abusers and non-abusers in having a family member during childhood who abused substances in the study by Fortunata and Kohn (2003), while perpetrators of IPV grew up in families with a lower socio-economic status (SES) than non-perpetrators in McKenry et al. (2006).

#### Substance Use

Addictive behaviors (Toro-Alfonso & Rodríguez-Madera, 2004) and substance use (i.e., both alcohol and other drugs use; Chong et al., 2013) were found to be both positively associated to IPV perpetration. However, in the study by Chong et al. (2013), substance use was related only to physical IPV, while the association with psychological IPV was not significant.

**Alcohol Use** Participants’ (Bartholomew et al., 2008a, b; Davis et al., 2016; Fortunata & Kohn, 2003; Kelley et al., 2014; McKenry et al., 2006; Schilit et al., 1990; Wu et al., 2015) or partner’s (Leone et al., 2022) alcohol use were found to be positively associated to IPV perpetration in eight studies. More specifically, in Davis et al. (2016), alcohol use was associated to physical/sexual and emotional IPV toward both regular and casual partner, and to controlling and HIV-related IPV perpetration toward regular, but not casual, partners. Monitoring IPV perpetration was not associated to alcohol use in this study (Davis et al., 2016). In addition, the relation between alcohol use and IPV perpetration was moderated by outness in Kelley et al. (2014): this association was significant only at high levels of outness. In Bartholomew et al., (2008a, b), the association between alcohol use and IPV perpetration was no longer significant when controlling for IPV victimization (i.e., bidirectionality of abuse). Physical aggression was associated to discrepant drinking between partners at a later time point in Lewis et al. (2018), while it was not related to subsequent physical aggression. Discrepant drinking was associated to subsequent psychological aggression and vice versa in this study (Lewis et al., 2018). Several studies did not find a significant association between alcohol use by the participants (Bacchus et al., 2017; Kelly et al., 2011; Reuter et al., 2015; Sharma et al., 2021; Stults et al., 2015b, 2021a) or their partners (Schilit et al., 1990; Sharma et al., 2021), and IPV perpetration. Alcohol dependence or abuse was associated to IPV in Fortunata and Kohn (2003), while this relationship was not significant in Bacchus et al. (2017).

**Drug Use** Drug use was related with IPV perpetration in seven studies (Bacchus et al., 2017; Bartholomew et al., 2008a, b; Fortunata & Kohn, 2003; Stults et al., 2015b, 2021a; Wong et al., 2010; Wu et al., 2015). However, in Wu et al. (2015), only methamphetamine use was associated to IPV perpetration, while marijuana, powdered or rock/crack cocaine, or heroin use were not. In addition, in Bacchus et al. (2017), participants who reported frightening and physically hurting their partner were at increased risk of cannabis use compared to those who did not. In contrast, there were no differences in cannabis use between those who perpetrate forcing sex or any abusive behaviors in the past 12 months, or those whose partner needs to ask permission to do activities, and those who did not. Furthermore, physically hurting a partner, but no other forms of abuse, was related with class A drugs (i.e., ecstasy, LSD, cocaine, crack, heroin, and injected amphetamines) use (Bacchus et al., 2017). In Bartholomew et al., (2008a, b), the association between drug use and IPV perpetration was no longer significant when controlling for IPV victimization. Drug use during sex was associated to IPV perpetration in two studies (Miltz et al., 2019; Wei et al., 2020a, b). In contrast, several studies did not identify a significant association between participants’ (Finneran et al., 2012; Kelly et al., 2011; Schilit et al., 1990; Sharma et al., 2021; Wei et al., 2021) or partner’s (Schilit et al., 1990; Sharma et al., 2021) drug use, and IPV perpetration.

#### Medical Conditions

Participants who reported to be HIV-positive were at increased risk of physical, but not emotional or sexual, IPV perpetration in the study by Stephenson et al., (2011a, b). In contrast, somewhat surprisingly, perpetrators of any abusive behaviors in the past 12 months were at lower risk of having a diagnosis of sexually transmitted infections (STI) than non-perpetrators in the study by Bacchus et al. (2017). Perpetrators of physical abuse, frightening, forcing sex, or controlling behaviors did not differ from those who did not perpetrate these forms of violence in the risk of having an STI diagnosis in this study (Bacchus et al., 2017). HIV status was not related with IPV perpetration in four studies (Bartholomew et al., 2008a, b; Finneran & Stephenson, 2014; Finneran et al., 2012; Stephenson & Finneran, 2016).

Furthermore, participants who perpetrated IPV did not differ from non-perpetrators in thinking their partner would not support their PrEP use or in not knowing if their partner would support their PrEP use, or in their perception of benefits provided by PrEP use in the study by Kahle et al. (2020).

#### Sexual Behaviors

**Years at Anal Sexual Debut and Sexual Partner(s)** Miltz et al. (2019) assessed the association between years at anal sexual debut and IPV perpetration, and they did not find significant results. In contrast, an age of 18 or older at sexual debut was positively associated to controlling behaviors and negatively related to emotional IPV in one study (Wei et al., 2020a, b). Wei et al. (2021) supported this latter finding, highlighting a negative association between age at sexual debut and IPV perpetration.

The number of sexual partners was positively associated to IPV perpetration in two studies (Zhu et al., 2021; Wei et al., 2020a, ). In Zhu et al. (2021), this association was moderated by self-efficacy (at high levels of self-efficacy the relation between multiple casual sexual partners, and IPV perpetration was no longer significant), while Miltz et al. (2019) did not find significant results. Behavioral bisexuality was not associated to IPV perpetration in two studies (Stephenson et al., 2011a; Finneran et al., 2012). Having group sex was associated to perpetration of lifetime, but not past year, IPV in one study (Miltz et al., 2019).

**Sexual Intercourses** Participants who reported two or more instances of anal receptive and insertive sex had a higher risk of perpetrating couple violence compared with those who reported no instances of these behaviors in the study by Stults et al. (2016). Use of lubrification was not associated to physical IPV perpetration in Stephenson et al. (2011a).

In addition, several studies assessed unprotected sex and IPV perpetration (Bogart et al., 2005; Finneran & Stephenson, 2014; Miltz et al., 2019; Stephenson & Finneran, 2017; Stephenson et al., 2011a; Stults et al., 2016). Most of these (five studies) found a relation between these two variables (Bogart et al., 2005; Finneran & Stephenson, 2014; Stephenson & Finneran, 2017; Stephenson et al., 2011a; Stults et al., 2016). However, in the study by Stephenson and Finneran (2017), condomless anal intercourse (CAI) was only associated to physical, sexual, emotional, and controlling IPV, while not to monitoring IPV. Furthermore, in the studies by Finneran and Stephenson (2014) and Stephenson et al. (2011a), perpetrators of physical IPV were more likely to have had unprotected anal intercourse than non-perpetrators of physical IPV. No differences in unprotected anal intercourse emerged between perpetrators of sexual IPV and non-perpetrators of sexual IPV. Furthermore, in Stephenson et al. (2011a), both sexual and physical IPV were higher among participants who have had unprotected anal intercourses (UAI) compared to those who have not, while in Finneran and Stephenson (2014) only for physical IPV the difference between the two groups was significant. Miltz et al. (2019) did not find significant associations between unprotected sex and IPV perpetration.

Inconsistent condom use with regular partner was related to any IPV and controlling violence perpetration in the study by Zhu et al. (2021), while this relation was not significant when considering physical, emotional, and sexual IPV. Inconsistent condom use with casual partners was instead only associated to sexual IPV (Zhu et al., 2021). Finally, experiencing transactional sex was associated to IPV perpetration in one study (Wei et al., 2021).

#### Sexual Minority-Specific Factors

The frequency of minority stressors experienced was not associated to pursuit behaviors and perpetration of negative behaviors after the breakup of the couple relationship in the study by Derlega et al. (2011).

**Experiences of Discrimination** Experiencing microaggressions (Taylor & Neppl, 2020) or homophobic discriminations or violence (Balsam & Szymanski, 2005; Finneran & Stephenson, 2014; Li et al., 2022; Swan et al., 2021; Swann et al., 2021; Whitton et al., 2021; Zavala, 2017) were found to be positively associated to IPV perpetration. The relation between microaggressions and IPV perpetration was moderated by sexual orientation (i.e., having a bisexual orientation increased the strength of the association between microaggressions and IPV perpetration) in the study by Taylor and Neppl (2020). Similarly, a moderating effect of commitment was found in the relation between homophobic discrimination and IPV perpetration: only at low levels of commitment in the relation this association remained significant. Furthermore, in Finneran and Stephenson (2014), the relationship between homophobic discrimination and IPV perpetration was no longer significant in the logistic model (only in the ANOVA test the differences between sexual batterers and non-batterers were significant). In the study by Balsam and Szymanski (2005), only lifetime discrimination was associated to psychological and physical/sexual (not LGB-specific abuse) IPV perpetration, while past-year discrimination was not. In Whitton et al., (2021), homophobic violence was associated only to psychological, and not physical or sexual IPV. Experiences of discrimination were not found to be associated to IPV perpetration in five studies (Ayhan Balik & Bilgin, 2021; Edwards & Sylaska, 2013; Finneran et al., 2012; Stults et al., 2021a; Zavala, 2017).

**Perceived Stigma** Perceived stigma was positively associated to IPV perpetration in four studies (Carvalho et al., 2011; Stults et al., 2015a, 2021a; Wei et al., 2020a, b). However, Stults et al. (2015a) found a positive association only between personal-local stigma and IPV perpetration, while the relation between public-gay related stigma and IPV was not significant. Furthermore, somewhat surprisingly, Stephenson et al., (2011a, b) found a negative association between sexual IPV perpetration and perceived local stigma-couple (i.e., perceived stigma around being in a same-sex relationship), but not with perceived local stigma-individual (i.e., perceived stigma around being a gay or bisexual man). No significant associations between perceived local stigma and physical or emotional IPV perpetration were found in this study (Stephenson et al., 2011a, b). Similarly, four studies did not identify associations between these variables (Edwards et al., 2021; Finneran et al., 2012; Stephenson et al., 2011a; Zavala, 2017); although in Edwards et al. (2021), this relation became significant at high levels of problem drinking, while it was not significant at low levels, social support did not moderate this relation.

**Internalized Homonegativity Toward Self and Others** Participants’ (Stephenson & Finneran, 2016) and partner’s (Turell et al., 2018) homo- or bi-negativity were found to be positively associated to IPV perpetration. McKenry et al. (2006) did not identify a significant association between IPV perpetration and family of origin’s homonegativity.

In addition, participants’ (Ayhan Balik & Bilgin, 2021; Balsam & Szymanski, 2005; Bartholomew et al., 2008a, b; Edwards & Sylaska, 2013; Finneran & Stephenson, 2014; Finneran et al., 2012; Kelley et al., 2014; Li et al., 2019, 2022; McKenry et al., 2006; Miltz et al., 2019; Pepper & Sand, 2015; Stephenson & Finneran, 2016; Suarez et al., 2018; Tognasso et al., 2022; Ummak et al., 2021; Zavala, 2017) and partner’s (Li et al., 2019) internalized homonegativity were found to be associated to IPV perpetration. However, in the study by Balsam and Szymanski (2005), internalized homonegativity was not related to psychological IPV and LGB-specific abuse, and the association between internalized homonegativity and physical/sexual violence was fully mediated by dyadic adjustment. Furthermore, sexual coercion perpetration was associated only with the religious attitudes toward lesbianism dimension of the Lesbian Internalized Homonegativity Scale (LIHS; Szymanski & Chung, 2001) in the study by Pepper and Sand (2015), while it was not related with any other dimension of the LIHS. Internalized homonegativity was not related with physical and emotional IPV perpetration in this study (Pepper & Sand, 2015) as in Ayhan Balik and Bilgin (2021), where only sexual IPV perpetration was positively associated to internalized homonegativity. In Finneran and Stephenson (2014), internalized homonegativity was associated to sexual IPV, but not with physical IPV perpetration, while Edwards and Sylaska (2013) found a significant relation between internalized homonegativity and physical and sexual IPV perpetration, but not between internalized homonegativity and psychological IPV. Participants’ and partner’s internalized homonegativity were only associated to psychological IPV in the study by Li et al. (2019), while no significant results were found when physical IPV perpetration was considered as the dependent variable. Similarly, Tognasso et al. (2022) identified a positive association between internalized homonegativity and any and psychological IPV perpetration, while the relation between internalized homonegativity and physical and sexual violence was not significant. In Li et al. (2022), the association between internalized homonegativity and partner’s psychological IPV perpetration (not participants’ psychological IPV nor participants’ and partner’s physical IPV) was moderated by commitment in the relationship: at high levels of commitment the relation became not significant. In this study (Li et al., 2022), commitment mediated the relation between internalized homonegativity and participants’ and partner’s physical and psychological IPV perpetration as well. In Finneran et al. (2012), internalized homonegativity was positively associated to sexual IPV perpetration only in the UK, while this relation was not significant in the USA, Canada, Australia, Brazil, or South Africa. Similarly, several studies did not find significant associations between participants’ (Carvalho et al., 2011; Chong et al., 2013; Edwards et al., 2021; Pistella et al., 2022; Whitton et al., 2021) and partner’s internalized homonegativity (Suarez et al., 2018) and IPV perpetration. In addition, in the study by Milletich et al. (2014), internalized homonegativity was not directly related with IPV perpetration. However, these authors found a positive indirect association between these variables that was mediated by fusion (Milletich et al., 2014).

**Sexual Identity Concealment** Outness was positively related to IPV perpetration in two studies (Ayhan Balik & Bilgin, 2021; Longares et al., 2018a). However, only overall outness, and not outness to religion and outness to family, were positively related with psychological IPV perpetration in Longares et al. (2018a). In Bartholomew et al., (2008a, b), outness was positively related with IPV perpetration when controlling for internalized homonegativity, though this relation became non-significant when controlling for both internalized homonegativity and violence receipt (i.e., bidirectionality of abuse). In contrast, Kelley et al. (2014) found lower levels of outness among IPV perpetrators compared with non-perpetrators. Outness was not related to IPV perpetration in four studies (Balsam & Szymanski, 2005; Carvalho et al., 2011; Edwards et al., 2021; Miltz et al., 2019).

**Gay Identity Development** Gay identity development was not related to IPV perpetration in the study by Stephenson et al. (2011a).

Finally, two studies elaborated conceptual models to understand the mechanisms through which minority stress contributes to IPV perpetration. Lewis et al. (2017) found a complex relation between discrimination, internalized homonegativity, perpetrator trait anger, perpetrator’s and partner’s alcohol problems, perpetrator’s relationship dissatisfaction, and psychological and physical violence. Similarly, in Mason et al. (2016), a complex relation between general life stress, distal and proximal minority stressors, negative affect, hazardous alcohol use, intrusiveness, and physical IPV perpetration was detected.

## Discussion

The current paper aimed to review and systematize the available literature on IPV perpetration among sexual minority people and its associated factors. Seventy-eight studies were included in the systematic review.

Several variables were found to be related with IPV perpetration among sexual minority people, and differences and similarities were found between IPV among heterosexuals and sexual minority people. Most of the assessed socio-demographic variables seem to not influence IPV perpetration in most of the included articles. Specifically, age, gender, gender identity, employment, and income were generally found to be unrelated to IPV perpetration. In contrast, when looking at differences across sexual orientations, bisexual people were at increased risk of IPV perpetration in several studies (Bermea et al., 2018; National Intimate Partner and Sexual Violence Survey, 2010). This result further underscores the double stigma associated to bisexual identity. The structural violence and the discrimination that seem to be conveyed by both the heterosexual and the lesbian and gay communities create additional stress and negative affect that can impact individual and relational wellbeing, ultimately leading to the perpetration of couple violence (Turell et al., 2018).

Many psychological factors were found to be related to IPV perpetration among sexual minority people. Depression was found to be related to IPV perpetration in several studies (Miltz et al., 2019; Sharma et al., 2021; Zavala, 2017). Two different theoretical perspectives can explain these findings. On the one hand, symptoms of depression can negatively influence coping and affect regulation mechanisms, which in turn can reduce the ability to manage conflicts and increase the likelihood of using violence toward the partner (Miltz et al., 2019). Accordingly, several studies identified an association between depression and relationship quality in both heterosexual (e.g., Morgan et al., 2018; Roberson et al., 2018) and sexual minority couples (e.g., Vencill et al., 2018; Whitton & Kuryluk, 2014). On the other hand, depression can be considered a consequence of IPV perpetration due to the psychological impact that this experience can entail (Sharma et al., 2021). Similarly, stress perception was associated to IPV perpetration in two studies (Craft et al., 2008; McKenry et al., 2006). Both individual and family stress can impact psychological wellbeing and produce negative affect that needs to be released even through violent behaviors (Zavala, 2017). In contrast, symptoms of anxiety, PTSD, and loneliness were generally unrelated with IPV perpetration (Bacchus et al., 2017; Stults et al., 2015a). Furthermore, several personality traits were found to be associated to IPV perpetration among sexual minority people. In particular, an abusive personality (Landolt & Dutton, 1997), hostility, emotional instability, and a negative worldview (Pepper & Sand, 2015) as well as higher scores on the aggressive (sadistic), antisocial, avoidant, passive-aggressive, self-defeating, borderline, paranoid, and schizotypal personality scale and on the alcohol-dependent, drug-dependent, bipolar (manic syndrome), and delusional clinical syndrome scales (Fortunata & Kohn, 2003) were associated to couple violence perpetration. Although only few studies assessed the association between these variables and other research are needed, these preliminary findings seem to equate those emerged among heterosexual couples (Brasfield, 2014; Brem et al., 2018; Gildner et al., 2021; Spencer et al., 2019), highlighting the need to consider personality traits in clinical settings. Self-esteem was negatively associated to IPV perpetration as well. These results are in line with the disempowerment theory of couple violence (Archer, 1994). According to this perspective, feelings of inadequacy and unworthiness, as well as lack of self-esteem can promote the use of violence to exert control over a partner who is perceived as threatening or who reveals their insecurities (Archer, 1994; McKenry et al., 2006). Between psychological factors, adult attachment seems to take a main role in predicting IPV perpetration among sexual minority people. Attachment theory conceived family violence as the result of dysfunctional strategies of distance and affect regulation (Bartholomew & Allison, 2006; Bowlby, 1984; Fonagy, 1999). Accordingly, attachment anxiety was found to be related to IPV perpetration in several studies (Bartholomew et al., 2008a, b; Derlega et al., 2011; Gabbay & Lafontaine, 2017a). High levels of attachment anxiety entail fears of rejection and loss, which can result in violence toward the partner as a form of exaggerated protest for their unmet attachment needs, driven by the use of strategies of hyperactivation of the attachment system (Bartholomew et al., 2008a, b; Gabbay & Lafontaine, 2017a). In contrast, conflicting results emerged regarding the association between attachment avoidance and IPV (Bartholomew et al., 2008a, b; Derlega et al., 2011; Gabbay & Lafontaine, 2017a, b). Although people with high levels of attachment avoidance can rely on IPV as a means of avoiding closeness and rejection (Gabbay & Lafontaine, 2017a), other studies are needed to confirm these hypotheses.

In addition to psychological factors, several relationship-level variables have also been found associated to IPV perpetration among sexual minority people. Specifically, while couple-level demographic factors (i.e., cohabitation, length of relationship, and having a child) were generally unrelated to IPV (Chong et al., 2013; Fortunata & Kohn, 2003; Sharma et al., 2021; Turell et al., 2018), relationship satisfaction/dyadic adjustment, conflict resolution skills, jealousy, and fusion/intrusiveness were associated to IPV perpetration in several studies (Balsam & Szymanski, 2005; Causby et al., 1995; Li et al., 2019; Mason et al., 2016; Milletich et al., 2014; Stephenson et al., 2011a, b; Telesco, 2003). These findings are in line with the model proposed by Bartholomew and Cobb (2011) to explain heterosexual IPV. As stated by the authors, regardless of personal dispositions to couple violence, those involved in mutually satisfying relationships, characterized by dyadic trust and a positive communication, are at lower risk of experiencing IPV. This theoretical perspective underlines the main role of stress within the couple as a predictive factor for IPV. Furthermore, considering the results found in several studies, lack of boundaries within the relationship (Causby et al., 1995; Mason et al., 2016; Milletich et al., 2014) and high levels of jealousy (Telesco, 2003) can promote IPV as well. In particular, people with high levels of enmeshment in their relationship can resort to abusive behaviors in order to restore a lost sense of oneness in the relationship following a partner’s attempt at separation, or conversely, to create a self-other distance when individuation and separateness are threatened. These data further highlight the role of dysfunctional mechanisms of interpersonal distance regulation in IPV perpetration (Bartholomew & Allison, 2006; Bartle & Rosen, 1994; Bowlby, 1984). In contrast, power dynamics within the couple seem to be unrelated to IPV perpetration among sexual minority people (Chong et al., 2013; Milletich et al., 2014).

Conflicting results emerged regarding the association between IPV perpetration and social- and community-level factors. Involvement in the LGBT community and support agencies was found to be positively related to IPV perpetration among sexual minority people (Wei et al., 2020a, b). Sexual minority people involved in the LGBT community have a greater likelihood to engage in social interactions, which in turn can increase the probability to perpetrate violence toward a romantic or sexual partner (Wei et al., 2020a, b). However, social support was generally unrelated to IPV perpetration (Reuter et al., 2015; Zavala, 2017). These findings are in contrast with several results emerged in studies conducted on heterosexual IPV (Gerino et al., 2018; Okuda et al., 2015; Richards & Branch, 2012), and further highlight the need to consider differences and similarities between these phenomena.

In line with these considerations, conflicting results emerged regarding the association between feminine and masculine gender expression, and IPV perpetration. While two studies identified a positive association between masculinity and IPV perpetration (Jacobson et al., 2015; McKenry et al., 2006), two other studies did not highlight significant results (Balsam & Szymanski, 2005; Telesco, 2003). The lack of significant results found in Telesco (2003) and Balsam and Szymanski (2005) demonstrated that the theory most commonly used to explain couple violence among heterosexual people, which conceives IPV as the result of endorsing a traditional masculinity which legitimizes the use of violence toward a subordinate partner (who exhibits feminine traits; Balsam & Szymanski, 2005; Telesco, 2003), may not be applicable within the LGB+ population (Balsam & Szymanski, 2005). However, these results could be influenced by methodological limitations (e.g., in Balsam and Szymanski (2005), only one item was used to assess masculinity and femininity), and other studies are necessary to understand the association between gender expression and gender role stereotypes, and the perpetration of IPV among sexual minority people.

Suffering violence in the family of origin was often found to be positively related to IPV perpetration among sexual minority people (Fortunata & Kohn, 2003; Schilit et al., 1991; Stults et al., 2015a; Toro-Alfonso & Rodríguez-Madera, 2004), although other studies did not confirm these results (Chong et al., 2013; McKenry et al., 2006; Milletich et al., 2014). From a psychoanalytic perspective, experiences of violence in the family of origin can result in feelings of unworthiness and in a lack of emotion regulation abilities, which can contribute to the use of violence within the relationship (Miltz et al., 2019). Furthermore, direct and indirect experiences of violence within the family of origin can serve as a model for conflict resolution that will be applied in future relationships (Zavala, 2017), according to the social learning theory (Felson & Lane, 2009; Gover et al., 2008; Mihalic & Elliott, 1997). However, considering the studies included in the current review, primarily suffering violence within the family of origin, rather than witnessing parental violence, emerged as a risk factor for IPV perpetration. Although further data are needed to confirm these findings, most of the data available to date suggest that only direct experiences of violence in the family of origin contribute to IPV in adulthood (Bartholomew et al., 2008a, b; McKenry et al., 2006; Milletich et al., 2014; Reuter et al., 2015).

Paralleling findings of studies conducted in heterosexual couples (see Cafferky et al., 2018 for a meta-analytic review), several articles included in the current systematic review identified a positive association between substance use and IPV perpetration among sexual minority people (Bacchus et al., 2017; Bartholomew et al., 2008a, b; Davis et al., 2016; Fortunata & Kohn, 2003; Kelley et al., 2014; McKenry et al., 2006; Miltz et al., 2019; Schilit et al., 1990; Stults et al., 2015b; Wei et al., 2020a, 2020b; Wong et al., 2010; Wu et al., 2015). As with other factors associated to IPV perpetration (e.g., mental health and couple satisfaction), two different theoretical perspectives can explain these findings. On the one hand, the psychoactive effect of drug and alcohol use and its neurological and psychological consequences can increase the risk of using violence to manage conflicts and stress within the relationship (Wei et al., 2020a, b). On the other hand, substance use can be conceived as a consequence of IPV perpetration. From this perspective, perpetrators of IPV can use substances to cope with the negative feelings related to the experience of couple violence (Lewis et al., 2018). A reciprocal relationship between these variables can exist as well (Lewis et al., 2018) and longitudinal studies are needed to confirm these hypotheses.

Furthermore, several studies found a positive association between sexual behaviors and IPV perpetration. Specifically, unprotected sex was related to IPV perpetration in five studies (Bogart et al., 2005; Finneran & Stephenson, 2014; Stephenson & Finneran, 2017; Stephenson et al., 2011a; Stults et al., 2016). As stated by several authors (Stephenson & Finneran, 2017; Stults et al., 2016), abusers can endorse a more stereotypical masculinity that promote impulsivity and hypersexuality, which can result in at-risk sexual behaviors. In contrast, medical conditions such as HIV-positive status do not seem to be associated to IPV perpetration in several studies (Bartholomew et al., 2008a, b; Finneran & Stephenson, 2014; Stephenson & Finneran, 2016).

While similarities emerged between IPV in heterosexual and sexual minority couples, as highlighted through the current systematic review, several studies identified factors specifically associated to couple violence among sexual minority people. These seem to be mainly related to the adverse conditions experienced by sexual minority people. Several dimensions of the minority stress model elaborated by Meyer (1995, 2003) were found to be associated to IPV perpetration among sexual minority people. In particular, while conflicting results emerged regarding the relation between IPV perpetration and experiences of discrimination, perceived stigma, and sexual identity concealment (e.g., Balsam & Szymanski, 2005; Bartholomew et al., 2008a, b; Carvalho et al., 2011; Edwards & Sylaska, 2013; Kelley et al., 2014; Longares et al., 2018a; Miltz et al., 2019; Taylor & Neppl, 2020; Wei et al., 2020a, b), internalized homonegativity was generally found to be associated to IPV (e.g., Balsam & Szymanski, 2005; Bartholomew et al., 2008a, b; Edwards & Sylaska, 2013; Kelley et al., 2014; Li et al., 2019; Miltz et al., 2019). Although it is likely that sexual minority people are better able to cope with distal minority stressors (Balsam & Szymanski, 2005), proximal minority stressors and in particular high levels of internalized homonegativity negatively impact self-esteem, self-worth, and self-identity, resulting in internal conflicts, a negative self-image, and feelings of fear and shame (Bartholomew et al., 2008a, b; Frost & Meyer, 2009; Kubicek et al., 2015; Meyer & Dean, 1998; Telesco, 2003). As suggested by several authors (Bartholomew et al., 2008a, b; Byrne, 1996; Cruz & Firestone, 1998), these negative affects toward the self and in particular toward one’s own sexual identity can be projected on the partner, resulting in IPV perpetration in order to destroy those negative parts of the self that have been expelled. In addition, the stress that can be associated to the status of sexual minority, and the accompanying emotional dysregulation (Hatzenbuehler, 2009; Sommantico & Parrello, 2021), seems to be regulated through the body by resorting to violent behaviors toward the partner. Accordingly, exploring the moderating role of mentalization or the mediating effect of emotional regulation abilities in the association between minority stress and IPV perpetration may further shed light on the complex dynamics that shape couple violence among sexual minority people. The application of the psychological mediation framework (Hatzenbuehler, 2009) which highlights the role of emotion regulation in the relation between sexual minorities stressors and wellbeing seems to show promising results in this direction.

Only few studies explored the mechanism through which minority stress influences IPV perpetration. Mediation models have demonstrated a complex relation between minority stress, couple-level variables, negative affect, alcohol problems, and IPV perpetration (Balsam & Szymanski, 2005; Lewis et al., 2017; Mason et al., 2016; Milletich et al., 2014). Specifically, internalized homonegativity seems to negatively affect relationship quality and couple dynamics, and increase negative affect and alcohol problems, increasing the probability to perpetrate IPV. The structural violence experienced by sexual minority people and the lack of social acceptance of non-heterosexual relationships (Balsam & Szymanski, 2005; Frost, 2011) seem to result in lower relationship quality, negative affect, and maladaptive behaviors which in turn promote IPV perpetration. More complex models are needed in order to further confirm these hypotheses.

## Limitations and Future Directions

When considering the results found in the current systematic review, several limitations need to be accounted for. First, this is not a meta-analysis, thus no statistical conclusion can be drawn.

Second, only data on the perpetration of IPV have been considered, and results on factors associated to IPV victimization among sexual minority people need to be explored in further reviews.

Third, only quantitative data were considered by design. Exploring results drawn from qualitative studies can provide a broader comprehension of the phenomenon and need to be considered in future studies.

Fourth, the population of our interest consisted of cisgender sexual minority people, while studies mainly conducted on gender minorities were excluded. Future reviews focused on factors associated to IPV among gender minorities are needed.

Finally, only original research papers published in English and indexed in the main psychological databases were included. Exploring results from other kind of sources such as reports from national and international institutions or NGOs, as well as studies published in languages other than English can deepen our understanding of IPV among sexual minorities people. In addition, methodological limitations emerged when considering the studies included in the current systematic review, which need to be considered in future studies to improve our understanding of IPV among sexual minority people.

Differences in the operational definitions of IPV and sexual orientation emerged, which can affect the results found and limit comparability between the studies. Although most of the included articles used validated assessment tools, many others evaluated at least some forms of IPV using items developed by the authors. In addition, only a few studies included questions about LGB+-specific abuse tactics. These methodological limitations negatively influence the opportunity to precisely detect couple violence among sexual minorities and need to be considered in future studies. The development of new tools aimed at assessing IPV among sexual minority people or the adaptation of instruments to date available for their use with this population are recommended.

Furthermore, differences were found between the included articles on criteria to enroll participants in the study. The various groups included under the umbrella-term sexual minority people can experience different forms of violence, and factors associated to IPV perpetration among self-identified LGB+ people can differ from those associated to IPV perpetrators among people who self-identify as heterosexual and report non-heterosexual sexual behaviors. How different definitions of sexual minority influence the results found among this broad population needs to be explored and controlled for in future studies.

Only few studies specified the type of relationship in which the violence occurred. This does not allow for firm conclusion regarding variables specifically associated to IPV in same-sex couples. How the type of relationship moderates or influences the results found within the studies aimed at assessing factors associated to IPV among sexual minority people needs to be considered.

In addition, only few studies used dyadic analysis techniques, and considering the interdependence between partners, future studies are needed to understand how characteristics of both partners can affect the risk of perpetrating IPV.

Moreover, all but one of the included studies have a cross-sectional design, which does not allow for firm conclusions about the causal direction of the associations found within the included studies. While drawing from different theoretical perspectives, many of the identified associated factors (e.g., adult attachment, personality traits, family of origin-related factors, and minority stress) are considered predictors rather than consequences of IPV perpetration. Longitudinal studies are needed to confirm these hypotheses.

In addition, the results of several included studies highlight a strong association between IPV victimization and perpetration, and a high occurrence of mutual violence among sexual minority people (e.g., Bartholomew et al., 2008a, b; Edwards & Sylaska, 2013). For these reasons, future studies need to assess both victimization and perpetration, and control for how they influence each other and the results found.

Only one study employed a cross-cultural design and most of the studies were conducted in the USA. Accordingly, other studies are needed to explore IPV perpetration and its associated factors in other geographic areas to fill these gaps.

Furthermore, only few studies were focused on ethnic minorities or people with a HIV-positive status. Drawing from an intersectional framework (Crenshaw, 1991), future studies should explore how multiple stigmatized dimensions of one’s own personal identity impact the risk of IPV perpetration among sexual minority people.

Finally, more complex models (e.g., mediational, moderation, or structural equation models) are needed to understand the mechanism through which minority stress, and psychological and relational factors are related to IPV perpetration among sexual minority people.

## Conclusions

The results of the current systematic review highlight the need to consider couple violence among sexual minority people through a multidimensional approach to account for the multitude of variables associated to IPV perpetration. On the one hand, conflicting results emerged regarding the association between gender expression and IPV perpetration. These findings show that the applicability of theories mainly used to understand IPV among heterosexual couples has not yet been demonstrated when considering IPV among sexual minority people. Other studies are needed to understand the role of adhering to traditional gender roles on IPV perpetration among sexual minority people.

On the other hand, the main role of psychological, relational, and LGB+-specific factors emerged in many of the included studies. Specifically, internal working models and adult attachment style, as well as high levels of stress, couple dissatisfaction and fusion within the relationship seem to play a major role in the perpetration of IPV among sexual minority people. These findings highlight the impact of negative affect, and dysfunctional mechanisms of interpersonal distance and affect regulation, and are in line with the conceptualization of couple violence provided by attachment theory (Bartholomew & Allison, 2006; Bowlby, 1984; Fonagy, 1999) and Bartle and Rosen (1994), which consider IPV as the result of dysfunctional strategies of self-other distance and affect regulation. These theoretical backgrounds allow for the overcoming of a gender-based conception of couple violence, fostering an understanding of violent phenomena beyond those typically perpetrated by men toward women in heterosexual relationships. This approach can guarantee the legitimacy of couple violence perpetrated and suffered by sexual minority people and enables the understanding of this complex phenomenon regardless of the gender or sexual orientation of the people involved.

In addition, the significant association found between minority stressors and IPV perpetration in many studies underlines the necessity to consider the structural violence experienced by sexual minority people and the stress that it entails as a possible explanation for the high levels of IPV identified in this population. In particular, internalized homonegativity and the negative affect it evokes need to be addressed in clinical settings, and prevention programs aimed at reducing social homonegativity and sexual stigma are needed to promote sexual minorities’ individual and relational wellbeing.

The identified results in the current systematic review highlight the importance of appropriate screening processes, able to identify variables that contribute to IPV perpetration for each single case. This allows referral to care-providers who are better suited to address the specific involved factors. A multidimensional approach able to consider the multitude of variables associated to IPV perpetration is necessary to prevent violent behaviors and promote the treatment of perpetrators, with the final aim to reduce relapses. The role of psychological and LGB+-specific factors, as well as relationship dynamics need to be considered for clinical purposes, to reduce IPV perpetration among sexual minority people.

Training of stakeholders working with couple violence or sexual minority people is needed to increase professional skills in dealing with IPV among sexual minority people and increase access to services, which is still limited by lack of awareness regarding this phenomenon and perceived stigma (Santoniccolo et al., 2021). The development of services and interventions based on empirical evidence, addressed to sexual minority perpetrators of IPV and able to take care of these complexities while adopting non-stigmatizing attitudes, is needed as well. The emerging results in the current systematic review can provide an updated guide to develop policies in this direction.

## Data Availability

Not applicable.

## Footnotes

1 Sexual minority people include all persons whose sexual identity, orientation or sexual behaviors differ from the majority of the society and the alleged norm (Math & Seshadri, 2013). In this paper, we will use interchangeably the term “sexual minority” or the acronym “LGB + ” to refer to lesbian, gay, bisexual, queer, questioning, unclear, and pansexual people, men who have sex with men and women who have sex with women.
